# Phosphodiesterase 10A (PDE10A) as a novel target to suppress β-catenin and RAS signaling in epithelial ovarian cancer

**DOI:** 10.1186/s13048-022-01050-9

**Published:** 2022-11-02

**Authors:** Rebecca M. Borneman, Elaine Gavin, Alla Musiyenko, Wito Richter, Kevin J. Lee, David K. Crossman, Joel F. Andrews, Annelise M. Wilhite, Steven McClellan, Ileana Aragon, Antonio B. Ward, Xi Chen, Adam B. Keeton, Kristy Berry, Gary A. Piazza, Jennifer M. Scalici, Luciana Madeira da Silva

**Affiliations:** 1 Gynecologic Oncology Division, Mitchell Cancer Institute, University of South Alabama, 1660 Springhill Avenue, Mobile, AL 36604 USA; 2 Department of Biochemistry and Molecular Biology, Center for Lung Biology, University of South Alabama College of Medicine, Mobile, AL USA; 3 Drug Discovery Research Center, Department of Pharmacology, Mitchell Cancer Institute, University of South Alabama, Mobile, AL USA; 4 Department of Genetics, University of Alabama at Birmingham, Birmingham, AL USA; 5 Cellular and Biomolecular Imaging Facility, Mitchell Cancer Institute, University of South Alabama, Mobile, AL USA; 6 Flow Cytometry Core Facility, Mitchell Cancer Institute, University of South Alabama, Mobile, AL USA

**Keywords:** PDE10A, Sulindac, β-catenin, RAS, Ovarian cancer

## Abstract

A leading theory for ovarian carcinogenesis proposes that inflammation associated with incessant ovulation is a driver of oncogenesis. Consistent with this theory, nonsteroidal anti-inflammatory drugs (NSAIDs) exert promising chemopreventive activity for ovarian cancer. Unfortunately, toxicity is associated with long-term use of NSAIDs due to their cyclooxygenase (COX) inhibitory activity. Previous studies suggest the antineoplastic activity of NSAIDs is COX independent, and rather may be exerted through phosphodiesterase (PDE) inhibition. PDEs represent a unique chemopreventive target for ovarian cancer given that ovulation is regulated by cyclic nucleotide signaling. Here we evaluate PDE10A as a novel therapeutic target for ovarian cancer. Analysis of The Cancer Genome Atlas (TCGA) ovarian tumors revealed PDE10A overexpression was associated with significantly worse overall survival for patients. PDE10A expression also positively correlated with the upregulation of oncogenic and inflammatory signaling pathways. Using small molecule inhibitors, Pf-2545920 and a novel NSAID-derived PDE10A inhibitor, MCI-030, we show that PDE10A inhibition leads to decreased ovarian cancer cell growth and induces cell cycle arrest and apoptosis. We demonstrate these pro-apoptotic properties occur through PKA and PKG signaling by using specific inhibitors to block their activity. PDE10A genetic knockout in ovarian cancer cells through CRISP/Cas9 editing lead to decreased cell proliferation, colony formation, migration and invasion, and *in vivo* tumor growth. We also demonstrate that PDE10A inhibition leads to decreased Wnt-induced β-catenin nuclear translocation, as well as decreased EGF-mediated activation of RAS/MAPK and AKT pathways in ovarian cancer cells. These findings implicate PDE10A as novel target for ovarian cancer chemoprevention and treatment.

**Supplementary Information:**

The online version contains supplementary material available at 10.1186/s13048-022-01050-9.

## Introduction

Ovarian cancer is the deadliest gynecologic malignancy, and the 5^th^ leading cause of all cancer deaths among women in the United States [1]. Less than half of all patients diagnosed with this malignancy survive past 5 years [1]. Despite advances such as risk-reducing surgery and treatment strategies including VEGF and PARP inhibitors, the obstacles of late diagnosis and treatment resistance remain drivers of its poor prognosis. Thus, there is a critical need for the identification of relevant targets with the potential to impact chemoprevention and treatment strategies to improve survival of this disease.

Nonsteroidal anti-inflammatory drugs (NSAIDs) have been well studied for cancer chemoprevention and show significant efficacy against multiple cancers [2, 3]. Several meta-analyses show significant risk reduction in ovarian cancer incidence among regular NSAID users, with one study reporting as much as a 20% risk reduction from daily aspirin use [4–6]. The application of NSAIDs for ovarian cancer chemoprevention is particularly relevant, because a leading theory of ovarian carcinogenesis implicates chronic inflammation from incessant ovulation as an oncogenic driver [7]. Monthly ovulatory cycles expose the ovary to a number of pro-inflammatory mediators, oxidative stress, and DNA damage, with frequent cycles of proliferation and repair, thus, increasing the likelihood of mutagenesis [7]. Further evidence supporting this theory is the significant risk reduction in ovarian cancer incidence among oral contraceptive users [8].

Unfortunately, side effects from long-term NSAID use can result in potentially fatal toxicities due to cyclooxygenase (COX) inhibition and suppression of physiologically important prostaglandins [9]. A large body of evidence, however, supports the possibility that the chemopreventive activity of NSAIDs is exerted through mechanisms unrelated to their COX inhibitory activity whereby these toxic side effects could be avoided by using derivatives lacking COX inhibitory activity but targeting such alternative mechanism [10]. For example, exisulind, the sulfone metabolite of the NSAID sulindac, is highly effective at decreasing cancer growth both *in vitro* and tumorigenesis *in vivo,* yet lacks COX-inhibitory activity [10].

Multiple studies report that the antineoplastic effects of exisulind and sulindac are exerted through phosphodiesterase (PDE) inhibition [11–14]. Several sulindac derivatives that lack COX inhibitory activity, but possess greater PDE inhibitory potency, were reported to exhibit potent activity in the inhibition of growth among multiple cancer cell lines [15–18]. There are eleven distinct PDE isozyme families which catalyze the breakdown of cyclic nucleotides, cAMP and/or cGMP. The inhibition of PDEs results in increased levels of cyclic nucleotides which activate protein kinases, PKA or PKG [19]. There is now an abundance of literature demonstrating that the inhibition of PDEs and increased cyclic nucleotide signaling results in powerful antineoplastic activity among many cancers, including ovarian [15–18, 20–24].

Recently, a novel non-COX inhibitory sulindac derivative, MCI-030 (aka ADT 061), was reported with high potency and selectivity to inhibit PDE10A and colon cancer growth. MCI-030 also showed strong chemopreventive activity in the *Apc*^+*/min−FCCC*^ mouse model of colorectal cancer [25]. Inhibition of PDE10A by either genetic silencing or pharmacological inhibition was also reported to decrease cancer cell growth through increased cGMP/PKG signaling and subsequent decreased β-catenin-dependent TCF/LEF transcriptional activity in colon and lung cancer cells [26, 27].

PDE10A is a dual substrate degrading phosphodiesterase that hydrolyzes both cAMP and cGMP [19]. Although PDEs and cyclic nucleotides play a critical role in the ovary, namely in regulating folliculogenesis, oocyte maturation, and ovulation, PDE10A has not been widely studied in this regard [28, 29]. The most notable research on PDE10A and ovulation comes from a bioinformatics analysis of microarrays in hCG stimulated mouse preovulatory follicles, where PDE10A was reported to be one of four essential genes in progesterone-related ovulatory networks [30].

Here, we demonstrate that PDE10A serves as a novel target for the treatment of ovarian cancer*.* Using PDE10A inhibitors and through the generation of PDE10A knockout (KO) ovarian cancer cells, we show that PDE10A inhibition decreases ovarian cancer cell growth by inducing cell cycle arrest and apoptosis. Furthermore, we demonstrate that these anti-cancer effects are exerted through elevated cAMP/PKA and cGMP/PKG signaling with subsequent decreased β-catenin oncogenic signaling, as well as decreased activation of the RAS/MAPK and PI3K/AKT pathways.

## Methods

### Reagents and drugs

Pf-2545920 was purchased from Selleck Chemicals (cat# S2687); KT-5823 (cat# 10,010,965) and H-89 (cat# 10,010,556) from Cayman Chemical; human recombinant EGF protein solution from ThermoFisher Scientific (cat# PHG0311L). MCI-030 was synthesized and characterized for purity and chemical structure by the Drug Discovery Research Center at our institution, as described previously [25]. Antibodies are described in Table S1.

### Cell lines and patient samples

Primary and immortalized ovarian surface epithelial cells, and ovarian cancer cell lines are outlined in Table S2. Cells were grown in a 37 °C humidified incubator with 5% CO_2_. All cell lines were expanded into numerous aliquots upon receiving and stored in liquid nitrogen; they were routinely and intermittently tested to be determined mycoplasma-free. Short tandem repeat DNA profiling was performed for cell line authentication by Genetica DNA Laboratories (LabCorp). The generation of OV-90 and SKOV3 PDE10A knockout cell lines is detailed in Supplemental Methods, Table S3 and Fig. S5. De-identified fresh-frozen ovarian tumors and normal ovary tissue samples were obtained from the Mitchell Cancer Institute (MCI) Biobank under a protocol approved by the institutional review board of The University of South Alabama (#723,194). Written informed consent from patients was obtained by the MCI Biobank in accordance with the Declaration of Helsinki. Cell treatment conditions, cell lysis, and patient tissue processing for qRT-PCR and western-blotting are described in Supplemental Methods.

### TCGA analysis

PDE10A mRNA expression and patient clinical survival data for ovarian cystadenocarcinoma patients of The Cancer Genome Atlas (TCGA) was downloaded using the cBioPortal [31, 32]. A detailed description of data analysis is included in Supplemental Methods.

### RNAseq of SKOV3 PDE10A knockout cells

Total RNA isolation from SKOV3 parental, empty vector clone 1B9 (EV 1B9), and two PDE10A knockout clones (KO 5H5 and 2F4) was performed using the TRIzol™ Reagent (Life Technologies) followed by DNase treatment with TURBO DNA-free kit™ (Life Technologies). Each group was sequenced with two biological replicates. mRNA-sequencing was performed on the Illumina NextSeq500 as described by the manufacturer (Illumina Inc., San Diego, CA) by the Heflin Center for Genomic Science Core Laboratories at the University of Alabama in Birmingham. Library prep, sequencing and data analysis are described in Supplemental Methods. Normalized counts data and STAR alignment statistics are provided in Supplemental Tables S13 and S22.

### Cell viability luminescence assay

Cells were seeded (1,000 cells / well) in 96-well black-wall clear-bottom tissue culture plates, followed by treatment with either compound or vehicle in 4 replicate wells for 72 h (37 °C, 5% CO_2_). Cell viability was measured with Cell Titer Glo Assay (Promega) as per manufacturer’s protocol. Luminescence reading was performed with a Synergy H4 Hybrid Reader (Biotek). Dose–response curves and IC_50_ values were calculated using a Sigmoidal dose–response (variable slope) on GraphPad Prism 8.

### Phenotypic *in vitro *assays

Description of assays performed to measure cancer cell tumor properties, such as cell proliferation, colony formation, cell cycle analysis, migration and invasion assays, is provided in Supplemental Methods.

### Xenograft mouse model

Animal studies were conducted in accordance with an approved protocol (#1,234,751) by the Institutional Animal Care and Use Committee of the University of South Alabama. Female athymic nude-Foxn1nu mice (Jackson Laboratory), 6 weeks of age, were injected intraperitoneally with 100 µl of cell suspension (2 × 10^6^ cells/mouse). The study was terminated and mice euthanized after 7 weeks, when greater than 20% of the mice in any group reached the following endpoints: signs of abdominal pain, hunched posture, weight loss of greater than 20% of starting body weight, weight gain exceeding 5 g and abdominal bloating (reflection of ascites and/or large tumors), decreased movement, anorexia, constipation and/or diarrhea. Euthanasia was performed in a CO_2_ chamber (fill rate of about 30% to 70% of the chamber volume per minute) followed by cervical dislocation. Tumor burden was assessed by measuring wet tumor weight, wet weight of affected organs, and volume of ascites. A detailed description of cell preparation and animal care can be found in Supplemental Methods.

### Cyclic nucleotide quantification and phosphodiesterase activity assay

Cells were serum starved overnight prior to treatments shown. Levels of cAMP and cGMP were measured using a competitive enzyme immunoassay (EIA) (Cayman Chemical or New East Biosciences) according to the manufacturers’ protocols. Cyclic AMP-phosphodiesterase activity was measured following a protocol described previously [33] and detailed in Supplemental Methods.

#### Statistics

Statistical analyses were performed using the Graphpad Prism software (La Jolla, CA). Comparisons between two samples used unpaired t-test, while those for more than two samples used one-way ANOVA, with the Dunnett’s test (multiple comparisons to a single control mean) or the Tukey’s test (multiple comparisons across all means). Kaplan–Meier curves generated for survival data were compared using the Logrank test. In all Figs., asterisks indicate: *p < 0.05, **p < 0.01, ***p < 0.001, ****p < 0.0001; “ns” denotes not significant.

## Results

### Clinicopathologic characteristics of PDE10A expression in human ovarian cancer

**Fig. 1:**
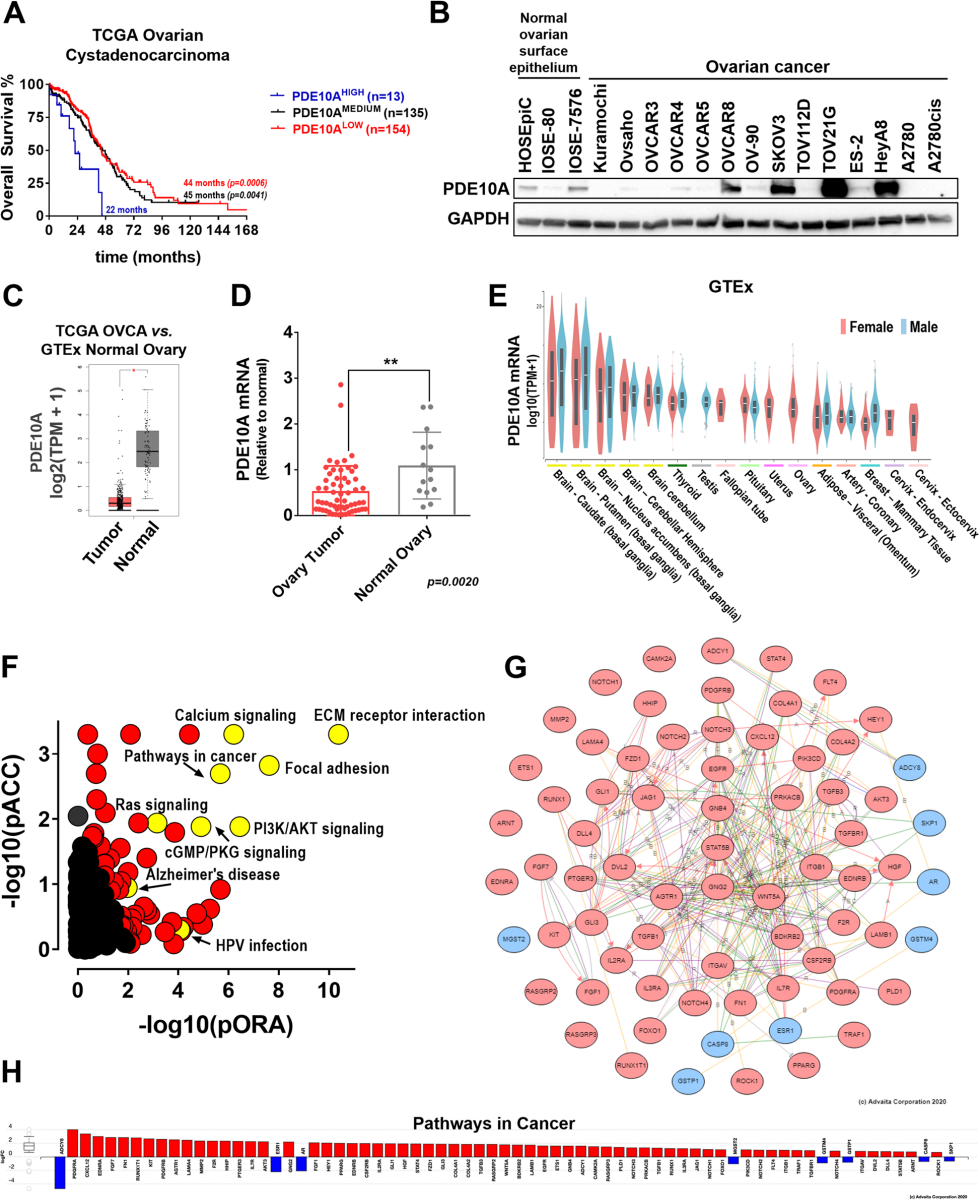
Clinicopathologic characteristics of PDE10A expression in ovarian cancer.** A** Kaplan-Meyer overall survival analysis of TCGA ovarian cystadenocarcinoma patients stratified by PDE10A mRNA expression levels. PDE10A^high^ and PDE10A^low^ correspond to mRNA expression 2x higher (*n* = 16) and 2 × lower (*n* = 154) than mean PDE10A mRNA levels in the population, respectively; PDE10A^medium^, remainder patients (*n* = 132). **B** PDE10A protein levels in ovarian cancer cell lines and normal ovarian surface epithelial (OSE) cells measured by western-blotting. GAPDH was used as the loading control. HOSEpic are primary OSE cells, while IOSE-80 and IOSE-7576 are immortalized OSE cell lines. **C** Comparison of PDE10A expression in normal ovary tissue (from GTEx database; *n* = 88) and ovarian cancer (from TCGA; *n* = 426) using GEPIA. **D** PDE10A mRNA levels measured by qPCR in ovary tumor (*n* = 59) and normal ovary (*n* = 14) clinical specimens deposited at our institutional Biobank. Relative expression was calculated with the ΔCt method using GPS1 as housekeeping gene. Error bars, ± SD, **p < 0.01 (Unpaired two-tailed students t-test). **E** PDE10A mRNA expression in normal human tissue from the GTEx Portal (top 16 tissues included). **F–H** iPathway Guide impact analysis of genes differentially expressed when comparing TCGA ovarian tumors with PDE10A^HIGH^ *vs.* PDE10A^LOW^ mRNA levels. In this comparison, 1,510 differentially expressed (DE) genes were identified out of a total of 19,331 genes with measured expression using thresholds of log2-fold change ± 0.6 and adjusted *p*-value < 0.1. **F** Each pathway is represented as a single dot, with significant pathways shown in red and yellow, and non-significant in black. **G** Hub genes and interactions for the Pathways in Cancer pathway. Genes with elevated mRNA expression depicted in red and genes with decreased mRNA expression depicted in blue. **H** Log2-fold change for the Pathways in Cancer gene set

We analyzed The Cancer Genome Atlas (TCGA) ovarian serous adenocarcinomas data to investigate clinical-pathological features associated with PDE10A. Somatic mutations in PDE10A were rare (3 out 606 samples), and 65.6% of the ovarian tumors showed decreased DNA copy number for PDE10A (Fig. S1A). PDE10A DNA gain and amplification was observed in 13.7% of the TCGA ovarian cancer patients, and this subset had significantly decreased disease-free survival when compared to the remainder of patients with heterozygous/homozygous loss or unaltered PDE10A copy number (11 *vs.* 18 months; *p* = 0.0417) (Fig. S1C-D). Gain of PDE10A DNA was correlated with a 3.5- and 2.6-fold increase in mean PDE10A mRNA expression compared to PDE10A heterozygous loss or diploid, respectively (Fig. S1B). When stratified by PDE10A mRNA levels, PDE10A^HIGH^ expressing ovarian cancer patients exhibited significantly worse overall survival (22 months) in comparison to those with low (44 months; *p* = 0.0006) or medium PDE10A expression (45 months; *p* = 0.0041) (Fig. 1A). Although not statistically significant, PDE10A^HIGH^ levels were weakly correlated with shorter disease-free survival (*p* = 0.1583) (Fig. S2A). Likewise, Prognoscan revealed a statistically significant shorter overall survival for patients with PDE10A^HIGH^ compared to PDE10A^LOW^ (34- *vs.* 86- months; *p* = 0.0159) in the Duke cohort of ovarian cancer patients (Fig. S2B) [34].

We then measured PDE10A protein levels in human ovarian cancer cell lines and compared with normal ovarian surface epithelial cells. Western-blot using a knockout validated PDE10A monoclonal antibody revealed that PDE10A protein expression was on average 28-fold upregulated in 4 out of 14 ovarian cancer cell lines (OVCAR8, SKOV3, TOV21G, and HeyA8), as compared to low or almost undetectable PDE10A protein in the remainder ovarian cancer cell lines and normal ovarian surface epithelial cells (Figs. 1B and S3A-B). PDE10A protein levels positively correlated with mRNA from sequencing data in the CCLE database (Pearson *r* = 0.7797; *p* = 0.0047) and from qRT-PCR (Pearson *r* = 0.9931; *p* < 0.0001) (Figs. S3C-D). A comparison of PDE10A expression levels in TCGA ovarian cancer samples and the GTEx database of normal tissue revealed that PDE10A mRNA expression was significantly lower in ovarian tumors as compared to normal ovary tissue (Fig. 1C). Clinical specimens obtained from our institutional biobank replicated this finding, showing two times higher PDE10A mRNA levels in normal ovary tissue as compared to ovarian tumors, and downregulation of PDE10A in tumors of 9 out 14 patients with matched normal ovary and ovary tumors (Figs. 1D and S3D). Finally, we looked at PDE10A expression in healthy tissues of female reproductive organs in the GTEx dataset. In agreement with previous literature reports, GTEx shows highest PDE10A mRNA levels in brain putamen and caudate, thyroid and testis [35]. Interestingly, female genital tract organs (fallopian tube, uterus, ovary, and cervix) were among the next highest PDE10A expressing tissues, as well as pituitary, omentum adipose tissue, coronary artery, and breast mammary tissue (Fig. 1E).

We also aimed to investigate the molecular pathways associated with PDE10A in the ovarian tumor microenvironment. First, we identified genes moderately to strongly coexpressed with PDE10A in the TCGA ovarian tumors based on Spearman correlation values provided in the cBioportal (Table S5). Pathway analysis investigating these gene lists in the Molecular Signatures Database (MSigDB) revealed several immune (cytokine-cytokine interaction, chemokine signaling), oncogenic (focal adhesion, MAPK, Jak-STAT, TGF-β, and Wnt signaling), and hormone-related (GnRH signaling and progesterone-mediated oocyte maturation) pathways that were positively correlated with PDE10A (Fig. S4A,C; Tables S6,S8,S10). Conversely, ribosome, spliceosome, oxidative phosphorylation, DNA replication, mismatch and base excision repair were among the main pathways identified for genes negatively correlated with PDE10A (Fig. S4B,C; Tables S7,S9,S11). We further investigated the molecular profile of PDE10A^HIGH^ *vs.* PDE10A^LOW^ expressing ovarian tumors using the mRNA comparison tool in the cBioportal (Table S12). Impact analysis revealed 66 pathways were significantly impacted including ECM receptor interaction, focal adhesion, PI3K/AKT signaling, RAS signaling pathway, MAPK signaling pathway, cGMP/PKG signaling, and TGF-β signaling pathway (Fig. 1F-H; Table S13). Network analysis and gene expression data, shown in Figs. 1G and H, highlight the many oncogenic-related targets that are upregulated in high expressing PDE10A ovarian tumors.

Altogether, these data suggest that PDE10A might play a role in normal physiology of the ovaries and female reproduction, and that upregulation of PDE10A in the context of ovarian cancer is associated with poor disease prognosis. In addition, the molecular profile associated with PDE10A-^HIGH^ expressing ovarian tumors suggests a microenvironment enriched with immune infiltrates, pro-inflammatory cytokines and chemokines, and the activation of many oncogenic signaling pathways.

### PDE10A gene knockout shows potent anti-tumor effects

**Fig. 2:**
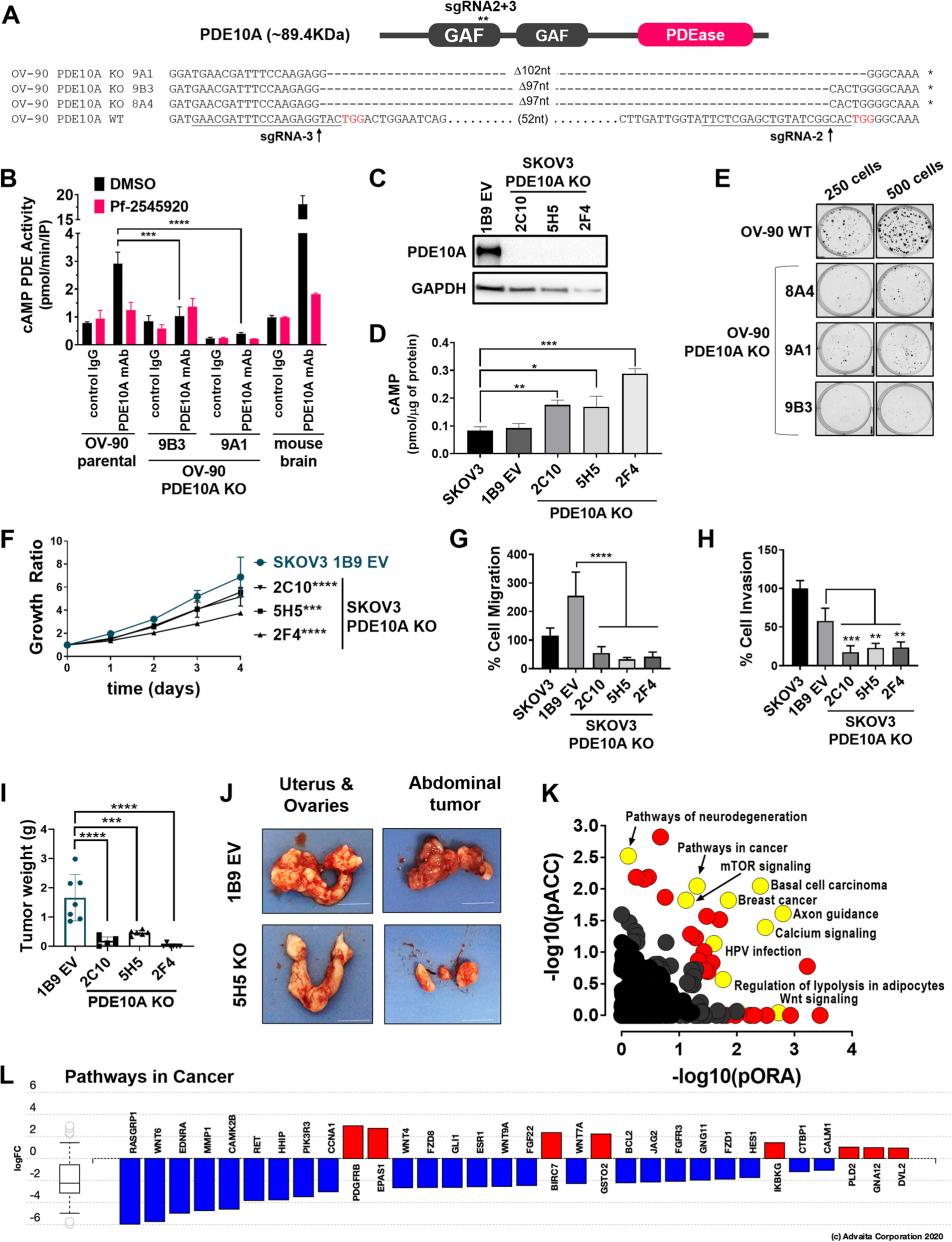
Antineoplastic effects of PDE10A gene knockout (KO) in ovarian cancer cells. **A** On top, schematic representation of PDE10A protein showing two cyclic nucleotide binding GAF domains at the N-terminus, and a phosphodiesterase catalytic domain (PDEase) at the C-terminus. On bottom, the genomic locus of PDE10A exon 7 was targeted by two CRISPR/Cas9 guide RNAs (sgRNAs). The predicted Cas9 cleavage sites are located 97 bp apart in exon 7. Successful deletion of the intended DNA fragment in OV-90 PDE10A KO clones in comparison to wild-type (WT) parental cells was confirmed by PCR followed by Sanger DNA sequencing. **B** PDE10A enzymatic activity in OV-90 parental and PDE10A KO clones was evaluated by immunoprecipitation of PDE10A followed by a cAMP-phosphodiesterase activity assay. Mouse brain tissue lysate was used as positive control. Pf-2545920 treatment was used to confirm that the measured activity was PDE10A-specific. **C** Confirmation of successful PDE10A KO in SKOV3 clones by western blotting. GAPDH was used as the loading control. **D** Baseline cAMP levels detected by ELISA in SKOV3 parental and PDE10A KO cells. Statistical significance was also observed when comparing KO clones to EV 1B9 control cells. Error bars, ± SD, ***p < 0.001, **p < 0.01, *p < 0.05 (student’s t-test). **E** Colony formation assays in OV-90 WT and PDE10A KO clones after 12 days of growth. **F** Proliferation of SKOV3 PDE10A KO clones. Growth ratios calculated by normalizing to day-0 seeding densities. p-values represent statistical significance on day 4. Error bars, ± SD, ***p < 0.001, ****p < 0.0001 (2-way ANOVA). **G-H** Migration (**G**) and invasion (**H**) of SKOV3 cells and PDE10A KO clones in the Boyden chamber assay over 24 h. Error bars, ± SD, ****p < 0.0001 (Ordinary one-way ANOVA). **I** Total tumor burden of athymic nude mice injected i.p. with SKOV3 PDE10A KO clones (*n* = 6 per group) or SKOV3 empty vector (EV 1B9) control cells (*n* = 7). Tumor burden included total tumor weights from injection site, uterus and ovary, and mesentery or abdominal tumors. ***p < 0.001, ****p < 0.0001 (Ordinary one-way ANOVA). **J** Representative images of uterus and ovaries and abdominal tumors from control and PDE10A KO nude mice groups. Scale bar = 1 cm. **K** iPathway Guide impact analysis of RNAseq results of DESeq2 differentially expressed genes comparing SKOV3 PDE10A KO clones (5H5 and 2F4) *vs.* SKOV3 parental and empty vector (1B9) control cells. In this comparison, 833 differentially expressed (DE) genes were identified out of a total of 14,195 genes with measured expression using thresholds of log2-fold change ± 0.6 and adjusted *p*-value < 0.05. Each pathway is represented as a single dot, with significant pathways shown in red and yellow, and non-significant in black. **L** Log2-fold change expression for “Pathways in Cancer” gene set. Upregulated genes are depicted in red and downregulated genes are depicted in blue

To investigate a functional role of PDE10A in ovarian cancer cells, we used CRISPR/Cas9 gene editing to generate PDE10A homozygous knockout (KO) clones in two ovarian adenocarcinoma cell lines, OV-90 and SKOV3 SKOV3 PDE10A KO clones were easily confirmed by western-blot given the strong expression of PDE10 in the parental cell (Fig. 2C). Conversely, this approach is less suitable for OV-90 cells, which exhibit only minor PDE10 expression and thus show only weak PDE10A-immunoreactive bands in Western blots even in parental cells. Therefore, we used PCR with primers flanking the sgRNA predicted sites from genomic DNA followed by Sanger DNA sequencing to confirm successful homozygous PDE10A knockout clones for OV-90 cells (Figs. 2A and S6). In addition, we used immunoprecipitation (IP) with PDE10A antibodies followed by measurement of cAMP-phosphodiesterase (PDE) activity recovered in IP pellets, as a sensitive biochemical approach to validate functional knockout clones [36]. As shown in Fig. 2B, PDE10-specific activity, defined as PDE activity sensitive to inhibition by the PDE10-selective inhibitor Pf-2545920 [37], was detected in parental OV90 cells, but was absent in the PDE10A-KO clones. IP from mouse brain, which is known to express high levels of PDE10, was used as positive control.

As expected, baseline cAMP was significantly elevated in all SKOV3 PDE10A KO clones compared with either empty vector control (EV 1B9) or parental cells (Fig. 2D). Colony formation, proliferation, and migration and invasion assays were also performed to study the effects of PDE10A gene KO on malignant properties of ovarian cancer cells. OV-90 PDE10A KO clones demonstrated a substantially decreased ability to form colonies as compared to WT OV-90 cells (Fig. 2E). Similarly, all three SKOV3 PDE10A KO clones exhibited significantly decreased proliferation by 4 days of growth compared to EV 1B9 control cells (Fig. 2F). Migratory and invasive capabilities of SKOV3 PDE10A KO clones were also significantly reduced compared to EV control and parental cells (Fig. 2G-H). *In vivo* tumorigenicity of SKOV3 PDE10A KO cells was assessed in an athymic nude xenograft mouse model 7 weeks after intraperitoneal implantation of cancer cells, which revealed significantly impaired neoplastic potential of PDE10A KO clones (Fig. 2I-J). The overall tumor burden of nude mice injected intraperitoneally with PDE10A KO cell lines was significantly reduced compared to vector control (Fig. 2I). Moreover, significant ascites was observed in two empty vector control mice (average volume = 2.25 ± 1.06 ml), but not in any of the PDE10A KO mice.

Contrasting the PDE10A-^HIGH^ expressing tumors from the TCGA, RNA sequencing of SKOV3 PDE10A KO cells revealed many oncogenic pathways downregulated in the PDE10A KO clones compared to their PDE10A-expressing control counterparts (Fig. 2K-L; Tables S14-S22). Genes in the Wnt pathway, breast cancer and basal cell carcinoma pathways were downregulated in SKOV3 PDE10A knockout cells. Furthermore, consistent with a physiological role for PDE10A in the brain striatum, axon guidance and pathways associated with neurodegeneration were among the most impacted by genetic disruption of PDE10A expression.

A meta-analysis comparison of the gene expression profile observed in the TCGA ovarian tumors expressing high levels of PDE10A with that of SKOV3 PDE10A KO cells revealed a number of genes, pathways, and biological functions overlap. Altogether, 89 differentially expressed genes overlapped between the two data sets, 53 of which showed inverse correlations coherent with PDE10A fold-changes in the two RNAseq datasets, including FZD1, GLI1, HHIP, and PDE3B (Fig. S6A-B; Table S23). Among overlapping GO biological functions affected by PDE10A, our meta-analysis identified sets of genes associated with anatomical structure morphogenesis, cell adhesion, cell motility, and cell migration (Table S24). Finally, a total of 13 pathways overlapped between the two datasets, including Alzheimer disease, breast cancer, cAMP signaling, cell adhesion molecules, gastric cancer, and proteoglycans in cancer (Fig. S6C; Table S25). Pathway diagrams reveal upregulation of a majority of genes in “Pathways in cancer” and “Breast cancer” for PDE10A-^HIGH^ expressing ovarian tumors from the TCGA (with both log-fold and perturbation analyses), whereas PDE10A KO clones show downregulation of many of these genes (Fig. S7).

Collectively, our data suggests that PDE10A modulates a variety of oncogenic molecular pathways to promote tumorigenic properties of ovarian cancer cells *in vitro* and *in vivo*.

### Inhibition of PDE10A with small molecule inhibitors confirms anti-cancer activity

**Fig. 3:**
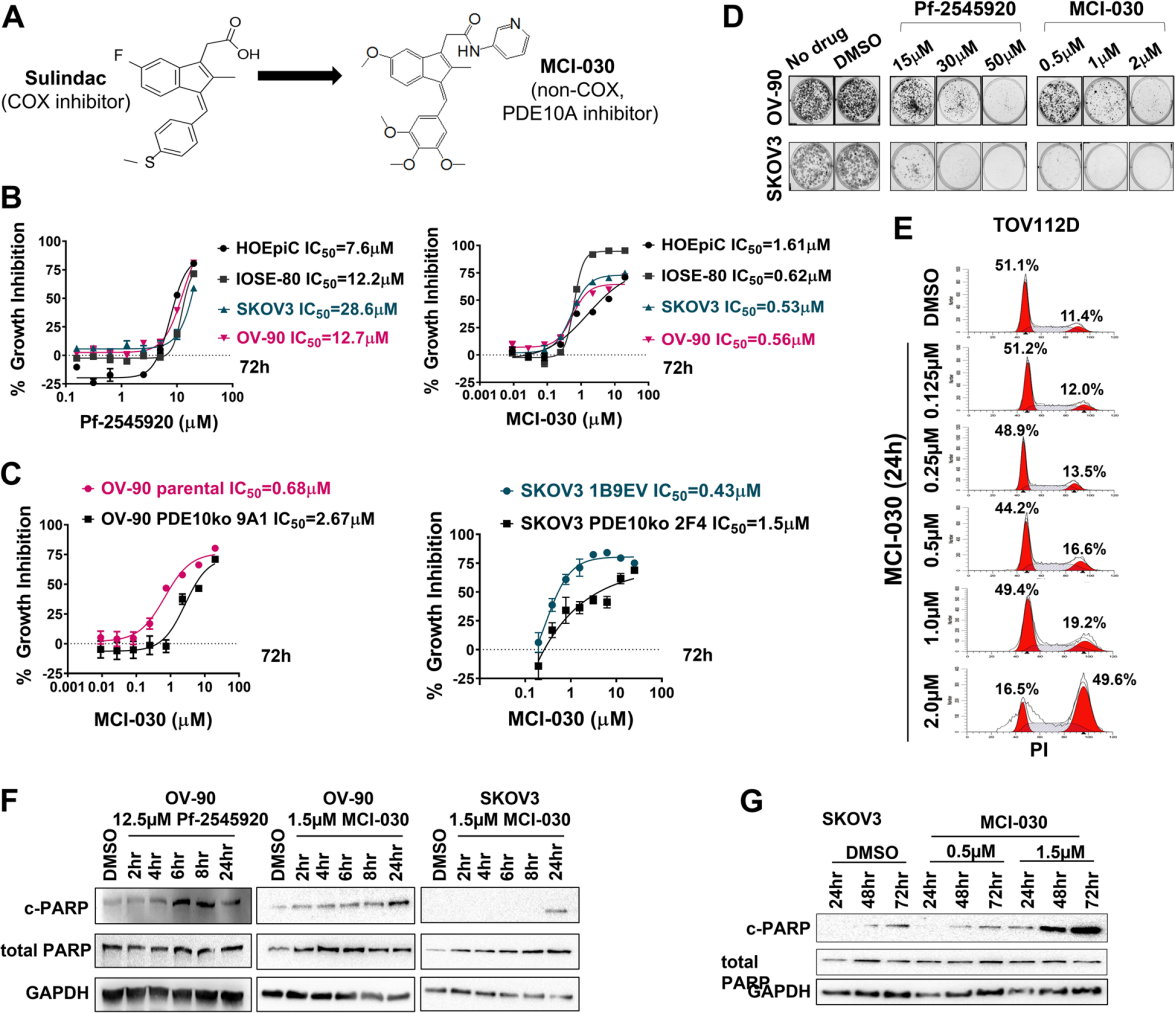
Anti-cancer effects of PDE10A inhibition in ovarian cancer cells.** A** Chemical structure for sulindac and its derivative compound MCI-030. **B-C** Dose-dependent growth inhibitory assays in ovarian cancer cells treated with Pf-2545920 and MCI-030 for 72 h measured by CellTiter-Glo. **D** Colony formation assays in OV-90 and SKOV3 cells treated with Pf-2545920 and MCI-030 for 12 days. **E** Cell cycle analysis in TOV112D cells treated with MCI-030 for 24 h using propidium iodide (PI) staining and flow cytometry cell sorting. **F-G** Time-dependent induction of PARP cleavage, a surrogate marker for apoptotic cell death, was assessed by western-blotting of OV-90 and SKOV3 cells treated with PDE10A inhibitors at increasing time intervals (drug concentration and incubation time as indicated). GAPDH was used as loading control

We tested two chemically distinct small molecule inhibitors, Pf-2545920 and MCI-030 (a.k.a. ADT 061) (Fig. 3A), to further assess various biological effects of PDE10A inhibition in various ovarian cancer cell lines and normal ovarian surface epithelial cells. First, we used a luminescent cell viability assay to evaluate the cell growth inhibitory effects of these compounds after 72 h treatment (Fig. 3B, Table S26). Pf-2545920, a highly selective and potent PDE10A inhibitor previously investigated in clinical trials for treatment of CNS disorders [37], inhibited growth of normal ovarian surface epithelial cells and various ovarian cancer cell lines with IC_50_ values ranging from 7.6 µM to 28.6 µM (Fig. 3B left panel, Table S26). These values are 4 orders of magnitude higher than the half maximal inhibitory concentration for this compound to inhibit PDE10A enzymatic activity, which suggests that other phosphodiesterases could also be inhibited [37]. MCI-030, a sulindac derivative recently reported as a novel PDE10A inhibitor with chemopreventive efficacy in the *Apc*^+*/min−FCCC*^ mouse model of colon cancer [25], exhibited more potent anticancer activity, with IC_50_ values ranging from 0.53 µM to 0.56 µM in ovarian cancer cells (Fig. 3B right panel; Table S26). These growth inhibitory IC_50_ values of MCI-030 are in line with those for enzyme activity inhibition previously described [25]. Notably, there was a fivefold decreased sensitivity to MCI-030 in the primary human ovarian surface epithelial cell line (HOSEpiC; IC_50_ = 3.03 µM) compared to ovarian cancer cells. Likewise, when evaluating growth inhibitory activity of MCI-030 in OV-90 and SKOV3 PDE10A knockout cells, we observed that knockout clones exhibited decreased sensitivity to MCI-030 compared to their control counterparts (2.7 to 5.8-fold and 2.3 to 4.0-fold, respectively) (Fig. 3C; Table S26).

We next evaluated the cell growth inhibitory effects of these compounds by colony formation assay, cell cycle analysis, and assessment of PARP cleavage as surrogate of apoptotic cell death. Colony formation assays showed that after 12 days of treatment with either MCI-030 or Pf-2545920, OV-90 and SKOV3 cells grew substantially fewer colonies compared to controls, an effect that was dose-dependent (Fig. 3D). Cell cycle analysis revealed that 24-h treatment of TOV-112D cells with MCI-030 resulted in cell cycle arrest in a dose-dependent manner, with a 40% increase in cells in the G2 phase with 2 µM MCI-030 compared to DMSO control (Fig. 3E). Finally, time-course experiments with Pf-2545920 and MCI-030 showed time-dependent induction of apoptosis, as measured by cleaved PARP detection by western-blotting. OV-90 cells showed a peak of PARP cleavage after 6 h of treatment with 12.5 µM Pf-2545920 and 24 h treatment with 1.5 µM MCI-030 (Fig. 3F). An extended treatment time course in SKOV3 cells revealed that maximum apoptosis was induced after 48 and 72 h of MCI-030 treatment (Fig. 3G).

### Inhibition of PDE10A increases cyclic nucleotides and activates PKA and PKG

**Fig. 4:**
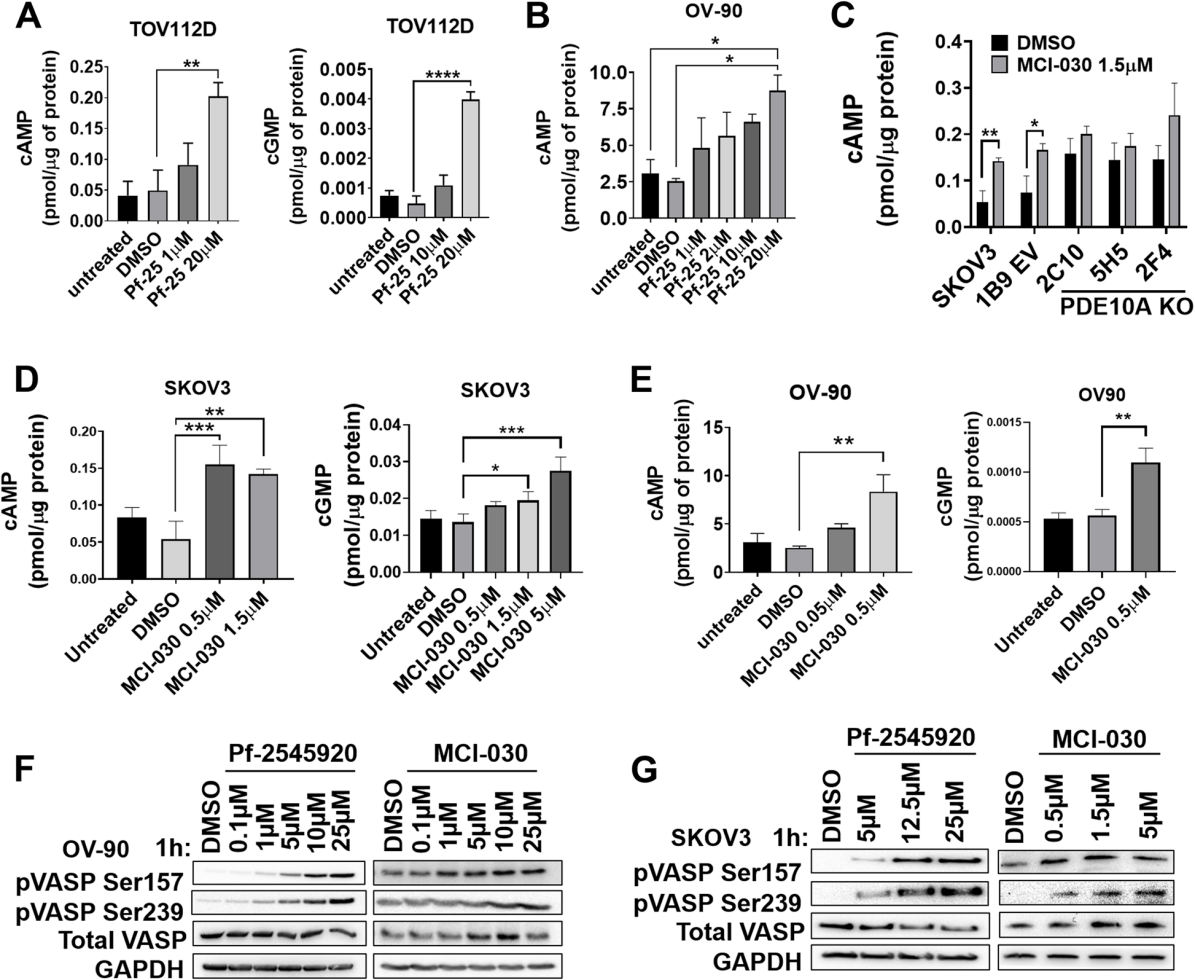
PDE10A inhibition in ovarian cancer induces cyclic nucleotide signaling.** A-B** Pf-2545920 increases cyclic nucleotide levels in a dose-dependent manner in TOV-112D (**A**) and OV-90 cells (**B**) (cAMP 1 h treatment, cGMP 1 h 30 min treatment). Error bars, SD, **p < 0.01, ****p < 0.0001 (Ordinary one-way ANOVA). **C** MCI-030 treatment for 30 min increases cAMP levels in SKOV3 parental and EV 1B9 cells, but not in PDE10A KO clones. cAMP levels were measured after cell treatment with 1.5 µM MCI-030 for 30 min. Error bars, SD; *p < 0.05, **p < 0.01 (unpaired Student’s t-test). **D-E** MCI-030 treatment for 30 min increases cAMP levels in a dose-dependent manner in SKOV3 (**D**) and OV-90 (**E**). Error bars, SD, **p < 0.01, ***p < 0.001 (Ordinary one-way ANOVA). **F** Treatment with PDE10A inhibitors for 1 h induces VASP phosphorylation at serine 157 (PKA site) and serine 239 (PKG site) in a dose-dependent manner in OV-90 and (**G**) SKOV3 cells. GAPDH was used as a loading control

To determine if the growth inhibitory activity of Pf-2545920 and MCI-030 were mediated by PDE10A inhibition and increased cyclic nucleotide signaling, cAMP and cGMP levels were measured by ELISA. TOV-112D cells treated with 20 µM Pf-2545920 showed significantly increased cAMP and cGMP compared to DMSO (Fig. 4A). Likewise, Pf-2545920 increased intracellular cAMP in OV-90 cells (Fig. 4B). OV-90 and SKOV3 cells also demonstrated significantly increased cAMP and cGMP levels with increasing concentrations of MCI-030 (Fig. 4D-E). Notably, the concentrations at which cyclic nucleotide levels were elevated are equivalent to each compounds IC_50_ values and the concentrations at which apoptosis and cell cycle arrest were induced. Interestingly, MCI-030 increased cAMP only in SKOV3 parental and EV 1B9 control cells, but not in the PDE10A KO clones, supporting a PDE10A mediated mechanism of action (Fig. 4C).

Vasodilator Stimulator Protein (VASP), a known downstream target of PKA and PKG, was used as a marker for protein kinase activation. VASP is preferentially phosphorylated at Ser^157^ by PKA and Ser^239^ by PKG [38–40]. As expected for a PDE10A inhibitor, treatment of OV-90 and SKOV3 cells with Pf-2545920 and MCI-030 for 1 h showed increasing phosphorylation of VASP at both Ser^157^ (PKA site) and Ser^239^ (PKG site) in a dose-dependent manner (Fig. 4F-G).

Altogether, our results show that Pf-2545920 and MCI-030 increase cAMP and cGMP and subsequent downstream activation of PKA and PKG, respectively, supportive of their proposed mechanism of action through inhibition of PDE10A.

### PDE10A inhibition elicits both PKA- and PKG-mediated signaling to promote apoptosis

**Fig. 5:**
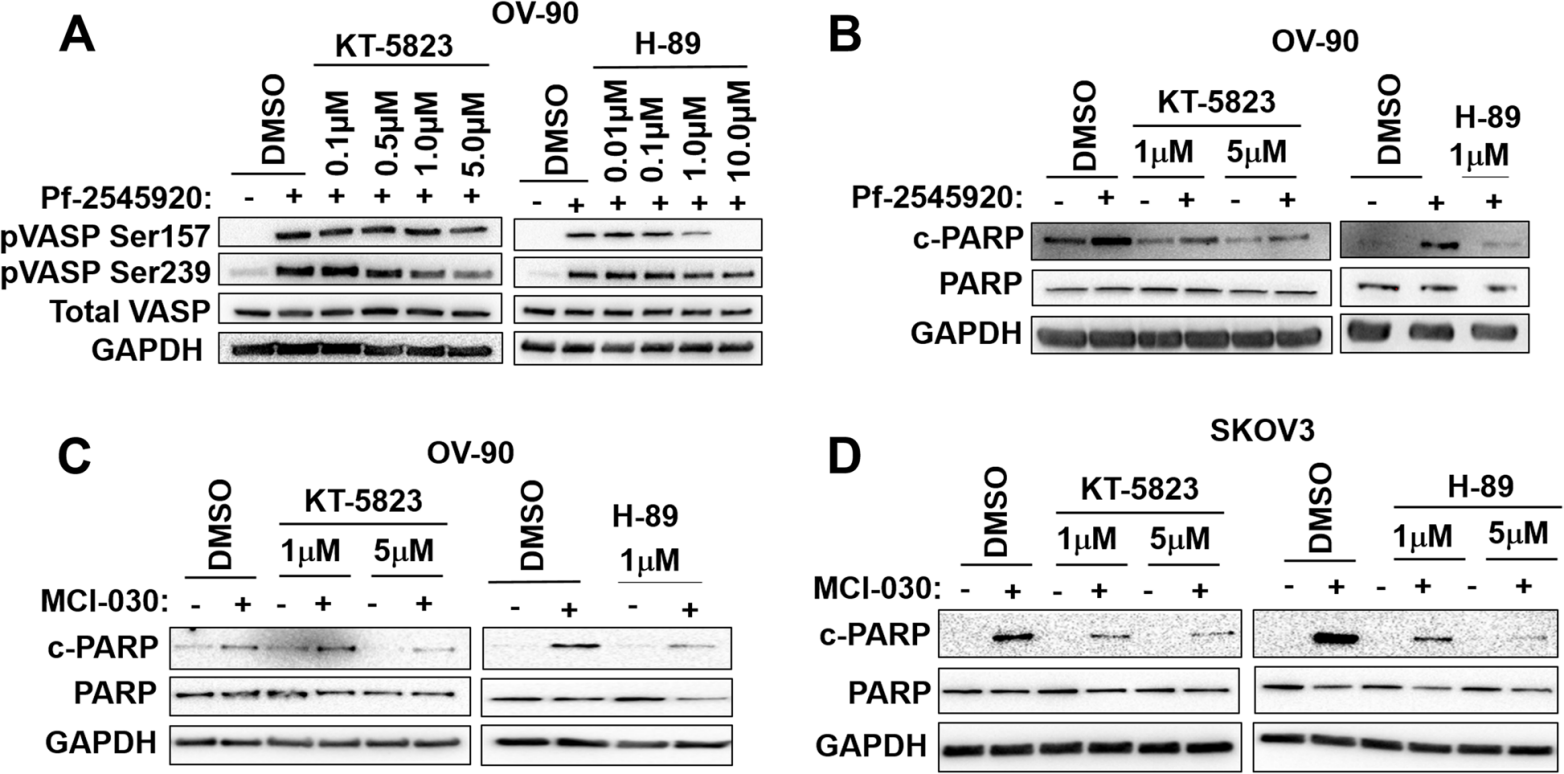
Apoptotic cell death induced by PDE10A inhibitors is mediated by PKA and PKG.** A** OV-90 cells pre-treated with the PKG inhibitor, KT-5823 (left), for 30 min or the PKA inhibitor, H-89 (right), for 10 min blocks Pf-2545920 activation of PKG and PKA, respectively, in a dose-dependent manner (Pf-2545920 treatment for 1 h). GAPDH was used as a loading control. **B** OV-90 cells pre-treated with the PKG inhibitor, KT-5823 (left), and the PKA inhibitor, H-89 (right), blocks Pf-2545920 induction of apoptosis (KT-5823 treatment for 30 min, H-89 10 min, Pf-2545920 treatment for 6 h). GAPDH was used as a loading control. **C-D** OV-90 and SKOV3 cells pre-treated with the PKG inhibitor, KT-5823 (left), for 30 min or the PKA inhibitor, H-89 (right), for 10 min blocks MCI-030 induction of apoptosis (MCI-030 treatment for 24 h). GAPDH was used as a loading control

To discern if PDE10A-induced PKA and PKG activation mediates cell death, we used the inhibitors H-89 and KT-5823 to block PKA and PKG signaling, respectively*.* First, we performed a dose-finding experiment in which OV-90 cells were pre-incubated with the kinase inhibitors at increasing concentrations and then PKA and PKG were activated through treatment with Pf-2545920. As shown in Fig. 5A, pre-treatment of OV-90 cells with 1 µM KT-5823 reduced the Pf-2545920-induced phosphorylation of VASP at Ser^239^ (PKG site) but not Ser^157^ (PKA site). Similarly, OV-90 cells pre-incubated with 1 µM H89 had a reduction of VASP phosphorylation at Ser^157^, but not Ser^259^ (Fig. 5A).

Next, we performed a similar experiment but probed for cleaved PARP to detect apoptosis. OV-90 cells were pre-incubated with KT-5823 and then treated with Pf-2545920 for 6 h, the time previously demonstrated to induce apoptosis (Fig. 5B). Pre-incubation of OV-90 cells with KT-5823 substantially reduced Pf-2545920-induced PARP cleavage, indicating that apoptosis induced by PDE10A inhibition is at least in part mediated by PKG activation (Fig. 5B left panel). Similar findings were observed for H-89, when pre-treatment with this PKA inhibitor substantially decreased Pf-2545920-induced PARP cleavage, demonstrating that PKA also mediates the apoptotic cell death induced by PDE10A inhibition (Fig. 5B right panel). Likewise, pre-treatment of OV-90 and SKOV3 cells with KT-5823 or H-89 substantially decreased MCI-030 induction of PARP cleavage (Fig. 5C-D). Thus, activation of both cAMP/PKA and cGMP/PKG signaling appear to be essential for the pro-apoptotic effects of PDE10A inhibitors.

### PDE10A inhibition decreases β-catenin, MAPK, and AKT oncogenic signaling in ovarian cancer cells

**Fig. 6:**
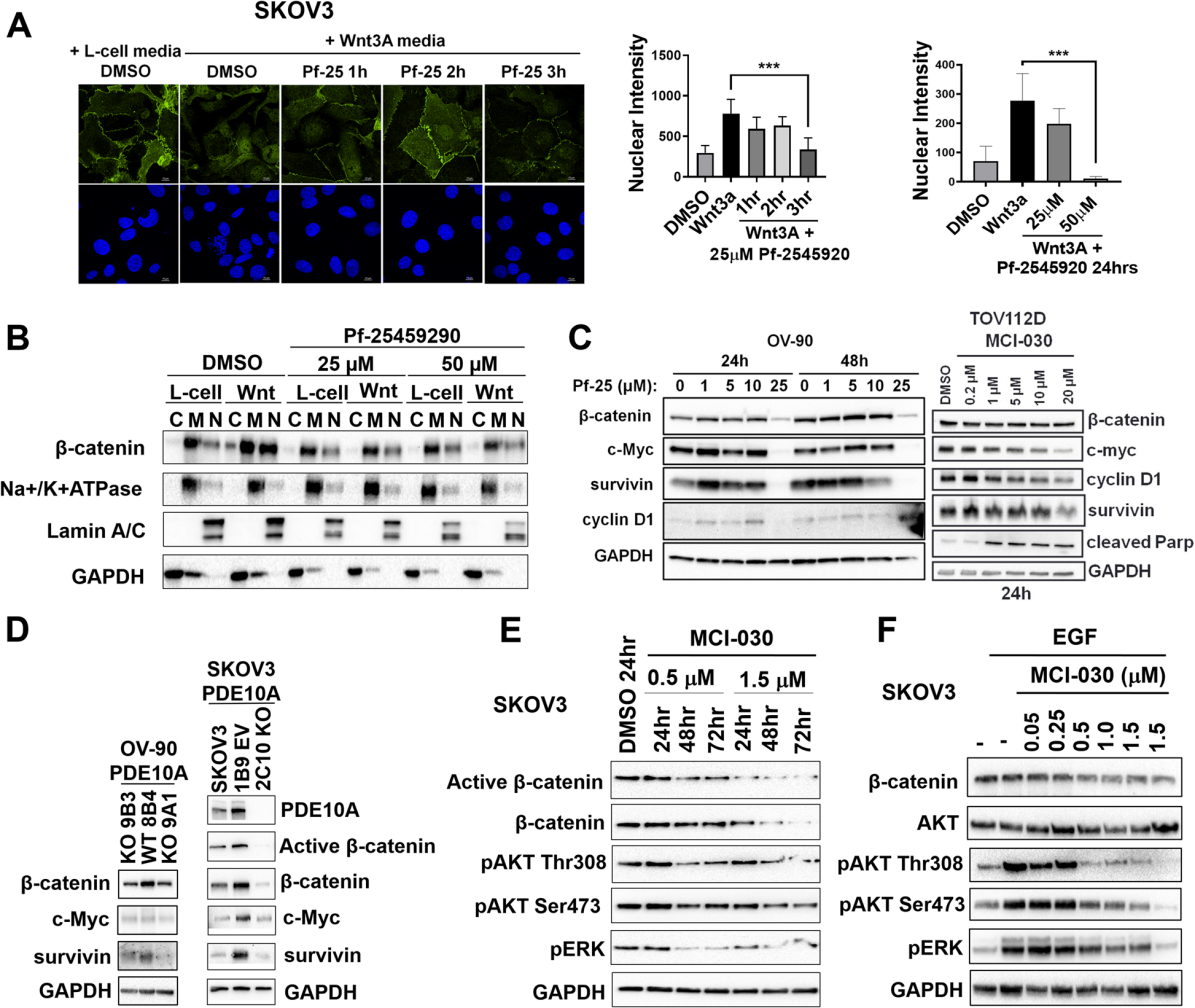
PDE10A inhibition decreases β-catenin, MAPK and AKT oncogenic signaling.** A** Left: confocal immunofluorescence microscopy of β-catenin and DAPI nuclear stain in SKOV3 cells pre-incubated with 25 μM Pf-2545920 or DMSO then treated with Wnt-3A conditioned media or L-cell media for additional 5 h; Right: quantitation of mean nuclear β-catenin intensity of experiment shown; Far right: quantitation of mean nuclear β-catenin intensity of SKOV3 cells pre-incubated with Pf-2545920 or DMSO for 24 h then stimulated with Wnt3A or L-cell media. Error bars, SD, (*n* = 3–7 images) ***p < 0.001 (ordinary one-way ANOVA) (**B**) Subcellular fractionation and detection of β-catenin in cytoplasmic [C], membrane [M], and nuclear [N] compartments of SKOV3 cells pre-incubated with Pf-2545920 or DMSO for 3 h then stimulated with Wnt-3A or L-cell media for additional 5 h. Loading controls were Na + /K + ATPase for membranous, Lamin A/C for nuclear, and GAPDH for cytoplasmic fractions. **C** Pf-2545920 and MCI-030 decrease β-catenin transcriptional targets in OV-90 and TOV112D cells. GAPDH was used as the loading control. **D** PDE10A gene KO decreases β-catenin levels and downstream transcriptional targets in OV-90 and SKOV3 cells. GAPDH was used as the loading control. **E** MCI-030 decreases activation β-catenin, MAPK, and AKT signaling pathways in SKOV3 cells in a time and concentration-dependent manner. GAPDH was used as the loading control. **F** Pre-incubation of SKOV3 cells with MCI-030 for 24 h inhibit EGF-induced MAPK and AKT signaling pathways (EGF treatment at 30 ng/mL for 5 min). GAPDH was used as the loading control

Previous studies have reported that PKG activation can inhibit the Wnt/β-catenin pathway by phosphorylation of β-catenin to induce ubiquitination and proteasomal degradation [41]. To determine if PDE10A inhibition and activation of cGMP/PKG signaling in ovarian cancer cells can decrease β-catenin oncogenic signaling, we measured nuclear translocation of β-catenin, which subsequently binds to TCF/LEF transcription factors to induce transcription of target genes that promote growth and proliferation [42, 43]. Translocation of β-catenin to the nucleus was induced using conditioned media from L-cells expressing the Wnt-3a ligand in SKOV3 cells pre-treated with Pf-2545920 or DMSO. Confocal microscopy showed decreased nuclear localization of β-catenin after 3 h of Pf-2545920 compared to DMSO (Fig. 6A). Similarly, 24-h Pf-2545920 pre-treatment followed by stimulation with Wnt-3a significantly decreased nuclear β-catenin (Fig. 6A). SKOV3 subcellular fractionation and detection of β-catenin levels in nuclear, cytoplasmic, and membranous compartments by western blot was also performed. In the absence of Wnt stimulation, β-catenin was predominantly in the membrane fraction; upon Wnt-3a stimulation, β-catenin became abundant especially in the nuclear compartment (Fig. 6B). Pre-incubation with Pf-2545920 or MCI-030 substantially decreased Wnt-3a induction of β-catenin nuclear translocation (Fig. 6B and S8).

The effect of PDE10A inhibitors on β-catenin activation of downstream TCF/LEF transcriptional targets was also investigated. Pf-2545920 treatment of OV-90 cells reduced total β-catenin and levels of TCF/LEF regulated genes, survivin, c-MYC, and cyclin D, in a time and concentration-dependent manner (Fig. 6C, left) [44–46]. Similarly, MCI-030 treatment of TOV-112D cells decreased total β-catenin as well as survivin, c-MYC, and cyclin D (Fig. 6C, right). OV-90 and SKOV3 PDE10A KO clones also exhibited decreased activation of β-catenin downstream targets including survivin and c-MYC, and the SKOV3 KO clone 2C10 showed substantially decreased levels of active and total β-catenin compared with controls (Fig. 6D).

PKA and PKG have also been shown to block RAS signaling to suppress the MAPK/ERK and PI3K/AKT pathways [47]. PDE10A inhibition in lung cancer was shown to decrease MAPK signaling [26]. Thus, we investigated if PDE10A inhibition in ovarian cancer cells blocks RAS signaling by measuring downstream effectors. SKOV3 cells treated with MCI-030 decreased baseline pERK levels at its activating phosphorylation sites, Thr^202^ and Tyr^204^, as well as decreased pAKT at its activating sites, Ser^473^ and Thr^308^ (Fig. 6E). Furthermore, pre-treatment of SKOV3 cells with MCI-030 attenuated EGF-stimulated RAS signaling in a dose-dependent manner, decreasing phosphorylation of ERK and AKT (Fig. 6F).

Altogether, our results show that PDE10A inhibition blocks three major oncogenic pathways in ovarian cancer cells, namely Wnt/β-catenin, MAPK and AKT signaling.

## Discussion

Our results establish PDE10A as a novel oncogenic mediator in epithelial ovarian cancer. While other studies have demonstrated that aberrant cyclic nucleotide signaling may contribute to ovarian cancer progression, the role of PDE10A in driving ovarian cancer has not been reported. Previous research has shown that cGMP/PKG signaling is downregulated in ovarian cancer compared to normal and preneoplastic tissues [48, 49]. Moreover, differential expression of the PKA catalytic subunits can drive ovarian cancer cell growth and differentiation [50]. Using small molecule inhibitors of PDE10A and genetic ablation of PDE10A expression we show that PDE10A-mediated regulation of both branches of cyclic nucleotide signaling – cAMP and cGMP – has potent anti-neoplastic effects including decreased cell proliferation, cell cycle arrest, and increased apoptosis in ovarian cancer cells. PDE10A knockout decreases tumorigenic properties of ovarian cancer cells such as migration, invasion and *in vivo* growth in athymic nude mice. We also demonstrate that PDE10A inhibition leads to decreased β-catenin oncogenic signaling and decreased activation of the MAPK and AKT pathways. Our findings are clinically relevant as they reveal that high expression of PDE10A in ovarian tumors correlates with significantly worse overall survival for patients and with upregulation of various oncogenic pathways, including Wnt and MAPK signaling, in the tumor microenvironment.

Contrary to the traditional upregulation of oncogenes in tumors when compared to normal tissue, we found that PDE10A mRNA expression is significantly decreased in ovarian tumors in comparison to normal ovaries, an observation based on qPCR data obtained with clinical specimens from our institutional cohort of ovarian cancer patients and confirmed with RNAseq data analysis of publicly available databases (TCGA and GTex). However, PDE10A upregulation was observed in some patient ovary tumors. Likewise, PDE10A was overexpressed in 4 ovarian cancer cell lines (OVCAR8, SKOV3, TOV21G, and HeyA8) in comparison to normal primary or immortalized ovarian surface epithelial cells, but most of the remainder 10 ovarian cancer cell lines showed very low levels of PDE10A protein expression as detected by western-blotting. These findings have led us to hypothesize two important aspects about PDE10A function in the normal and cancerous ovary. First, we speculate that the relative abundance of PDE10A in the normal ovary may be explained by the essential role of PDEs and cyclic nucleotide signaling in ovarian physiology. This is supported by the reproductive endocrinology literature, where PDE expression has been shown to change cyclically with ovulation and is dependent on hormonal signaling [29]. Gene expression in normal human organs in the GTEx database indicates that the ovary is among the top PDE10A expressing tissues. Future assessment of PDE10A expression throughout the ovulatory cycle is warranted and may shed insight on its role in regulating hormonal control of ovulation and explain its relative abundance in the normal ovary. Second, we hypothesize that PDE10A is not an oncogenic driver, but rather can impact the tumor microenvironment to drive cancer progression and malignancy. Our pathway analysis shows that Wnt and MAPK oncogenic signaling pathways are positively correlated with PDE10A expression as well as many other oncogenic pathways in the TCGA ovarian tumors*.* Thus, we propose that upregulation of PDE10A in ovarian cancer will lead to worse patient outcomes due to increased oncogenic signaling that provides cells with growth and survival advantages as well as a tumor microenvironment favorable for cancer progression.

PDE10A gene knockout in two ovarian cancer cell lines, SKOV3 and OV-90, confirmed the antineoplastic activity from targeting PDE10A. We observed significantly decreased proliferative ability and clonogenic potential in PDE10A KO clones, as well as a decreased ability to migrate and invade. RNA sequencing revealed a suppression of many oncogenic genes in PDE10A KO clones, including pathways in cancer more generally, and the Wnt pathway. β-catenin signaling was disrupted by PDE10A KO as evidenced by decreased levels of β-catenin and decreased activation of its downstream transcriptional targets in SKOV3 and OV-90 cells. Tumorigenicity of PDE10A KO clones was also significantly reduced *in vivo* as evidenced by xenograft mouse models. Importantly, we found an extensive overlap between the transcriptional profile of PDE10A knockout ovarian cancer cells and that of patient provided ovarian tumors expressing high PDE10A levels, which not only revealed several key genes and pathways directly impacted by PDE10A-mediated signaling *in vitro* but also those of importance in the patient-tumor microenvironment. Thus, we show an essential role of PDE10A in ovarian cancer cells to mediate multiple aspects of malignancy.

Pf-2545920, a well characterized highly potent and specific PDE10A inhibitor, and MCI-030, a novel PDE10A inhibitor that we recently reported to selectively inhibit colon cancer cell growth *in vitro* and adenoma formation in the *Apc*^+*/min−FCCC*^ mouse model of colon cancer [25], were found to selectively suppress ovarian cancer cell growth. Notably, MCI-030 displayed increased potency with IC_50_ values of approximately 0.5 μM – similar to what we reported for colon cancer cells [25] and approximately 15- to 60-fold higher than the values observed for Pf-2545920. The growth inhibitory activities of both MCI-030 and Pf-2545920 were associated with G2 cell cycle arrest and induction of apoptosis in ovarian cancer cells. Again, our results mirror those seen in colon and lung cancer cells in which PDE10A inhibition arrested the cell cycle, induced apoptosis, and inhibited cancer growth [26, 27, 51]. Hence, our *in vitro* data demonstrate the efficacy of PDE10A inhibitors as chemotherapeutics for ovarian cancer, warranting further exploration of their use in *in vivo* model systems.

We further demonstrate that PDE10A inhibition leads to an increase in both cAMP/PKA and cGMP/PKG signaling, and the activation of these protein kinases coincides with the growth inhibitory and apoptotic activity of Pf-2545920 and MCI-030 in ovarian cancer cells. Notably, these results differ from previous studies using PDE10A inhibitors in lung and colon cancers, which concluded that cGMP/PKG signaling alone was responsible for growth inhibitory activity of PDE10A inhibitors [26, 27, 51]. We postulate that this difference is due to the robust role of cAMP/PKA signaling within the ovary wherein it modulates the majority of gonadotropin signaling [29, 52]. cAMP is also more abundant in the ovary compared to cGMP*,* and PDE10A is thought to be a cAMP-inhibited cGMP PDE due to its higher K_m_ for cGMP (3 μM) compared to cAMP (0.05 μM) and a five-fold higher V_max_ for hydrolyzing cGMP [53, 54]. Alternatively, these differences could be explained by varying expression of other PDE isozymes and PKA/PKG subunits in ovarian cancer cells compared to colon and lung cancer cells.

Our results demonstrating that PKA and PKG are necessary for the apoptotic effects of PDE10A inhibition support several recent studies in ovarian cancer. Researchers targeting the cAMP specific PDE4 have demonstrated that elevated cAMP/PKA signaling increases apoptosis and decreases platinum resistance and enhances platinum sensitivity in ovarian cancer cells [22, 23]. Similarly, studies targeting and elevating cGMP/PKG signaling in ovarian cancer show that this can inhibit proliferation and induce apoptosis [55–57].

Activated cGMP/PKG signaling through PDE10A inhibition was shown to decrease β-catenin signaling in colon and lung cancers [26, 27, 51]. A number of previous studies demonstrated that PKG can inhibit the β-catenin pathway in several cancer types [41]. Our results support these findings. Namely, we show that PDE10A inhibition in ovarian cancer cells rapidly decreased levels of total and active β-catenin as well as the expression of downstream TCF/LEF regulated genes. We also demonstrate that PDE10A inhibition prevents canonical Wnt-stimulated nuclear translocation of β-catenin*.* Studies have found different mechanisms by which PKG can inhibit β-catenin signaling, thus, investigation into these inhibitory pathways should be explored to further elucidate the mechanism behind our findings [41].

Importantly, the Wnt/β-catenin pathway is known to play an important role in ovarian cancer. Higher β-catenin activity has been frequently documented in epithelial ovarian cancer, particularly in the high-grade serous subtype, and is thought to be due to dysfunction in a number of regulators of the pathway [58]. Additionally, numerous studies have shown a clear role that β-catenin signaling plays in maintaining stem-like properties in ovarian cancer which subsequently drives platinum resistance [58, 59]. Thus, our results point to a novel mechanism in abrogating this oncogenic pathway, which may prove particularly useful in the treatment of cisplatin resistant ovarian cancer.

Finally, we also show that PDE10A inhibition was able to decrease activation of both the MAPK and AKT pathways. Several previous studies in ovarian cancer have demonstrated that increased cGMP/PKG signaling decreases activation of both the MAPK and AKT pathways, and that this corresponds with decreased proliferation and increased apoptosis [55–57]. PDE10A inhibition in lung adenocarcinoma also lead to decreased activation of ERK and MEK [26]. Both PKA and PKG have been shown to directly inhibit components of the MAPK pathway leading to decreased pro-growth signaling [47, 60]. Further exploration into the mechanism by which PDE10A inhibition and increased cyclic nucleotide signaling interacts with ERK and AKT should be explored.

**Fig. 7:**
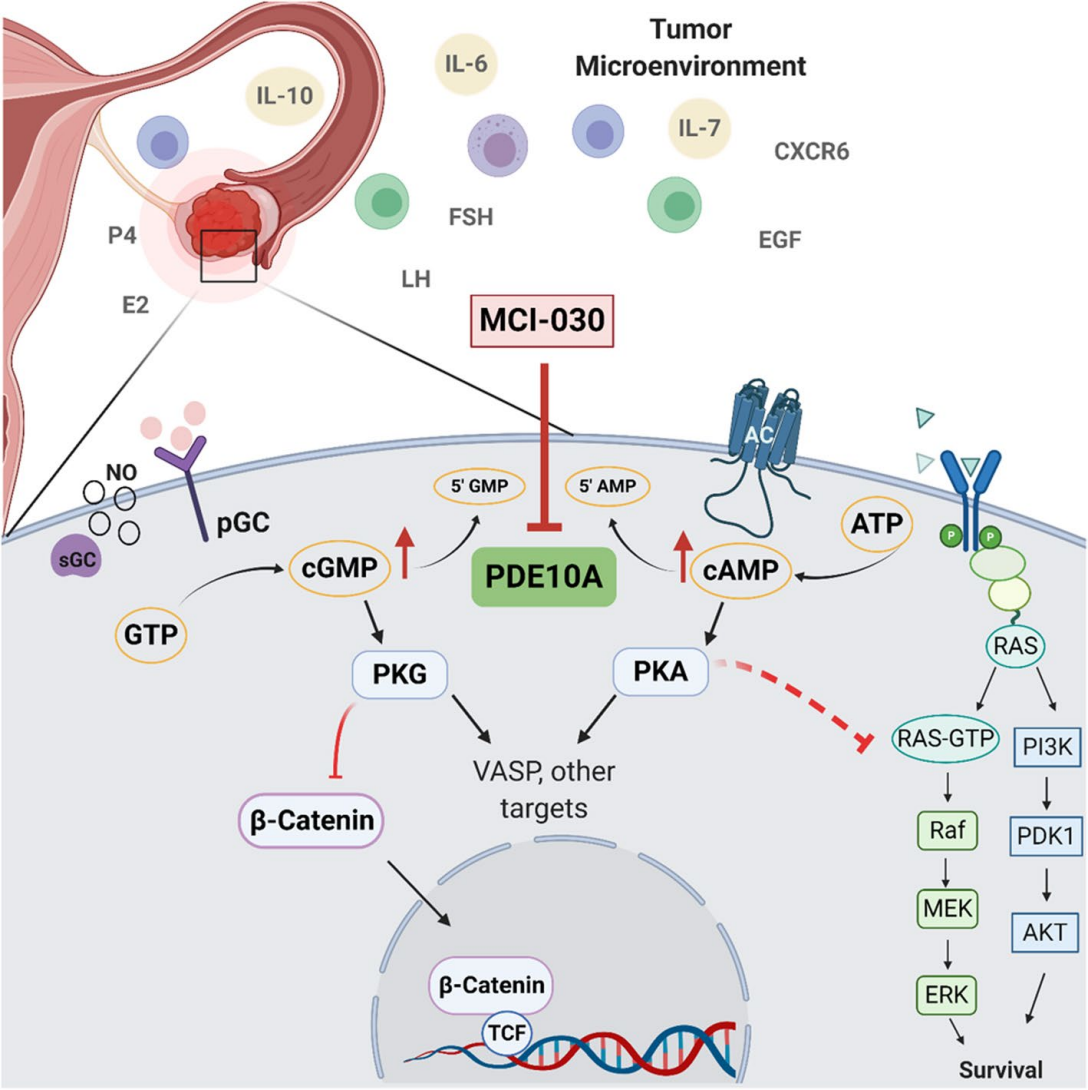
Schematic of proposed mechanism for PDE10A inhibition anti-tumor effects in ovarian cancer. Elevated PDE10A alters the tumor microenvironment by influencing hormonal and inflammatory signaling. MCI-030 inhibits PDE10A in ovarian cancer cells resulting in increased cAMP and cGMP levels and activation of protein kinases, PKA and PKG, respectively. PKG inhibits β-catenin, decreasing nuclear translocation and transcription of downstream TCF/LEF targets. PKA may inhibit RAS signaling decreasing ERK and AKT activation

In summary, our results show for the first time that PDE10A as a promising target for the treatment of ovarian cancer. Moreover, PDE10A expression in normal ovarian cells warrants its future investigation as a target during the early steps of carcinogenesis. As represented in the schematic Fig. 7, the antineoplastic effects of PDE10A inhibition involve an increase in cAMP/PKA and cGMP/PKG signaling, which leads to inhibition of β-catenin oncogenic signaling and decreased activation of the MAPK and AKT pathways. Altogether, our findings suggest a novel role for PDE10A in ovarian tumorigenesis, which could serve as a target for the chemoprevention or treatment of ovarian cancer.

## Supplementary Information

**Additional file 1. **
