# Supplementary material for: Phosphodiesterase 10A (PDE10A) as a novel target to suppress β-catenin and RAS signaling in epithelial ovarian cancer
Source: J Ovarian Res. 2022 Nov 2;15:120. doi: 10.1186/s13048-022-01050-9 (PMC9632086; doi:10.1186/s13048-022-01050-9)
Supplement: Supplementary file 1 — Additional file 1. [file 13048_2022_1050_MOESM1_ESM.zip › Supplemental Figure Combined.pdf]

Fig S1

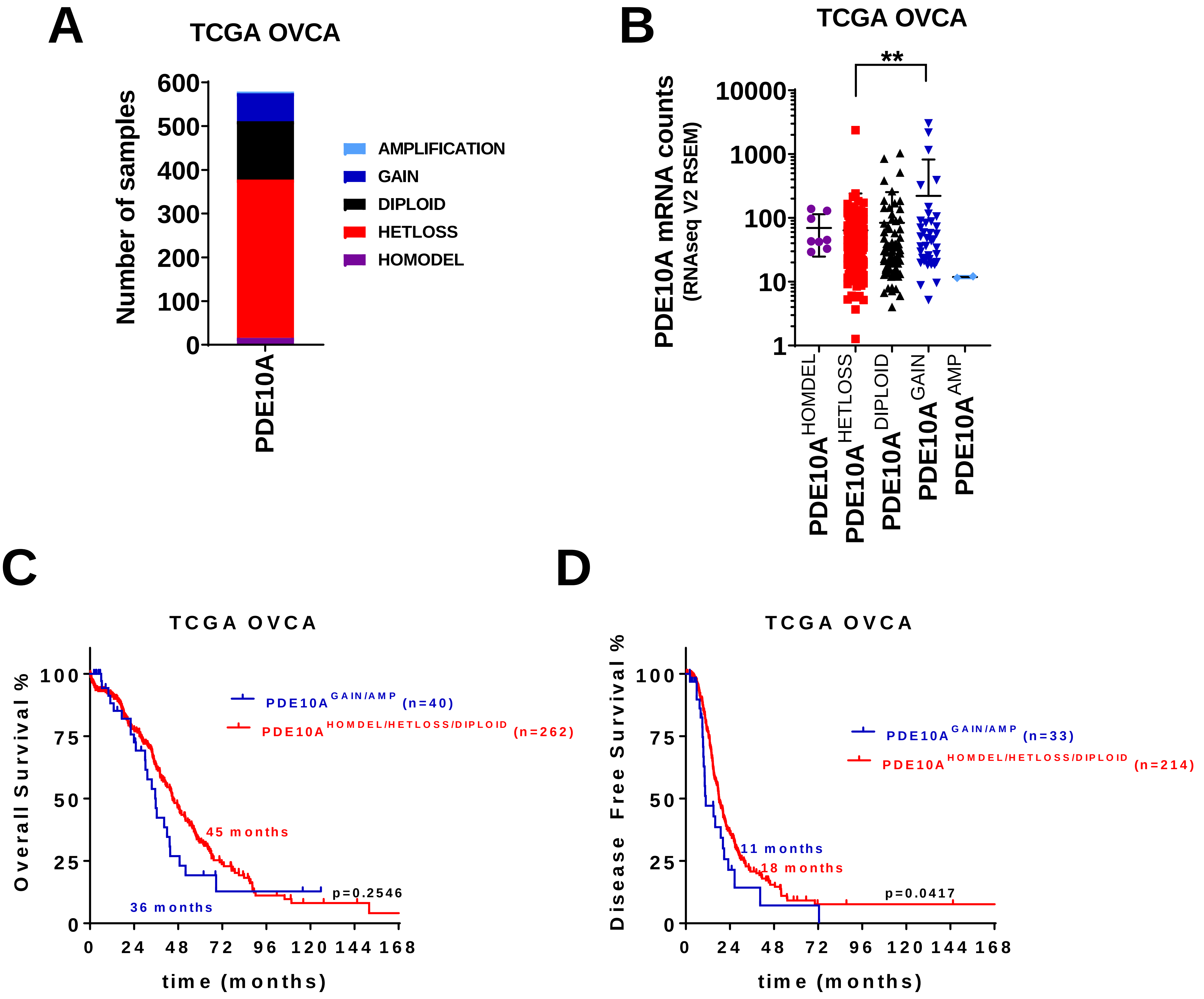
A
TCGA OVCA
Number of samples
600
500
400
300
200
100
0
PDE10A
AMPLIFICATION
GAIN
DIPLOID
HETLOSS
HOMODEL
B
TCGA OVCA
**
PDE10A mRNA counts
(RNAseq V2 RSEM)
10000
1000
100
10
1
PDE10A HOMDEL
PDE10A HETLOSS
PDE10A DIPLOID
PDE10A GAIN
PDE10A AMP
C
TCGA OVCA
Overall Survival %
100
75
50
25
0
PDE10A GAIN/AMP (n=40)
PDE10A HOMDEL/HETLOSS/DIPLOID (n=262)
45 months
36 months
p=0.2546
0 24 48 72 96 120 144 168
time (months)
D
TCGA OVCA
Disease Free Survival %
100
75
50
25
0
PDE10A GAIN/AMP (n=33)
PDE10A HOMDEL/HETLOSS/DIPLOID (n=214)
11 months
18 months
p=0.0417
0 24 48 72 96 120 144 168
time (months)

Fig S2

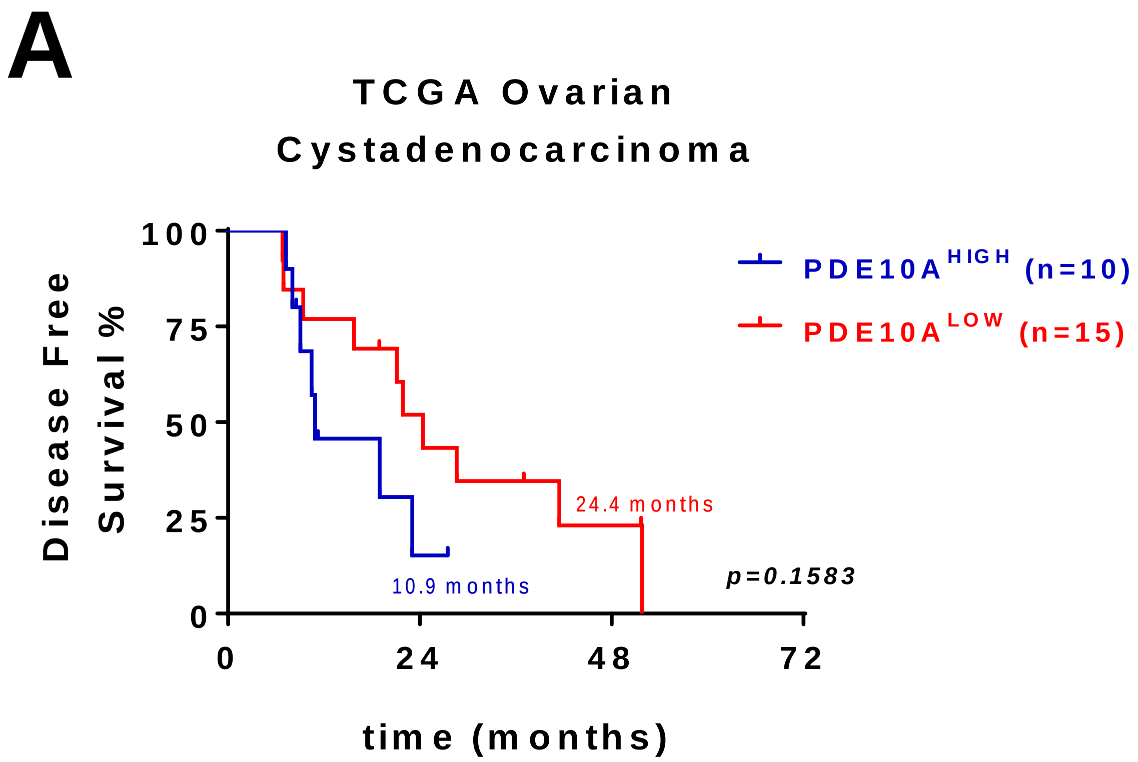
A
TCGA Ovarian
Cystadenocarcinoma
Disease Free Survival %
100
75
50
25
0
0
24
48
72
time (months)
PDE10A HIGH (n=10)
PDE10A LOW (n=15)
24.4 months
10.9 months
p=0.1583

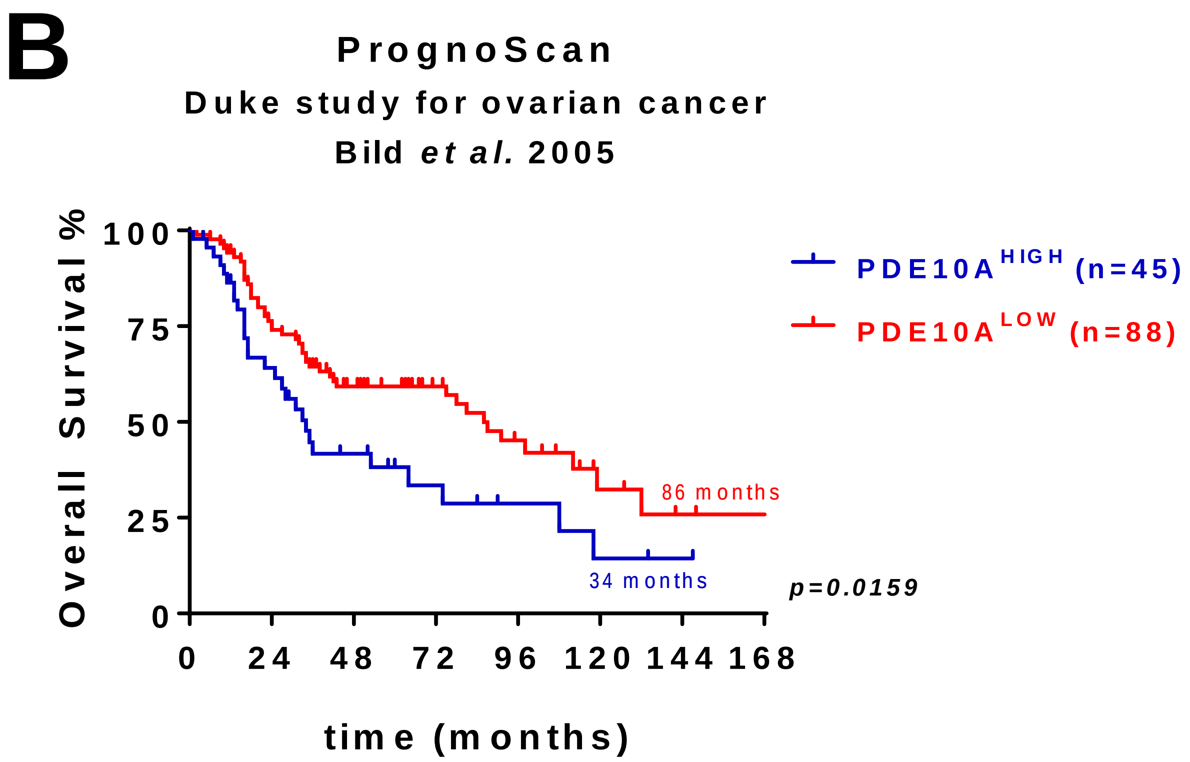
B
PrognoScan
Duke study for ovarian cancer
Bild et al. 2005
Overall Survival %
100
75
50
25
0
0
24
48
72
96
120
144
168
time (months)
PDE10A HIGH (n=45)
PDE10A LOW (n=88)
86 months
34 months
p=0.0159

Fig S3

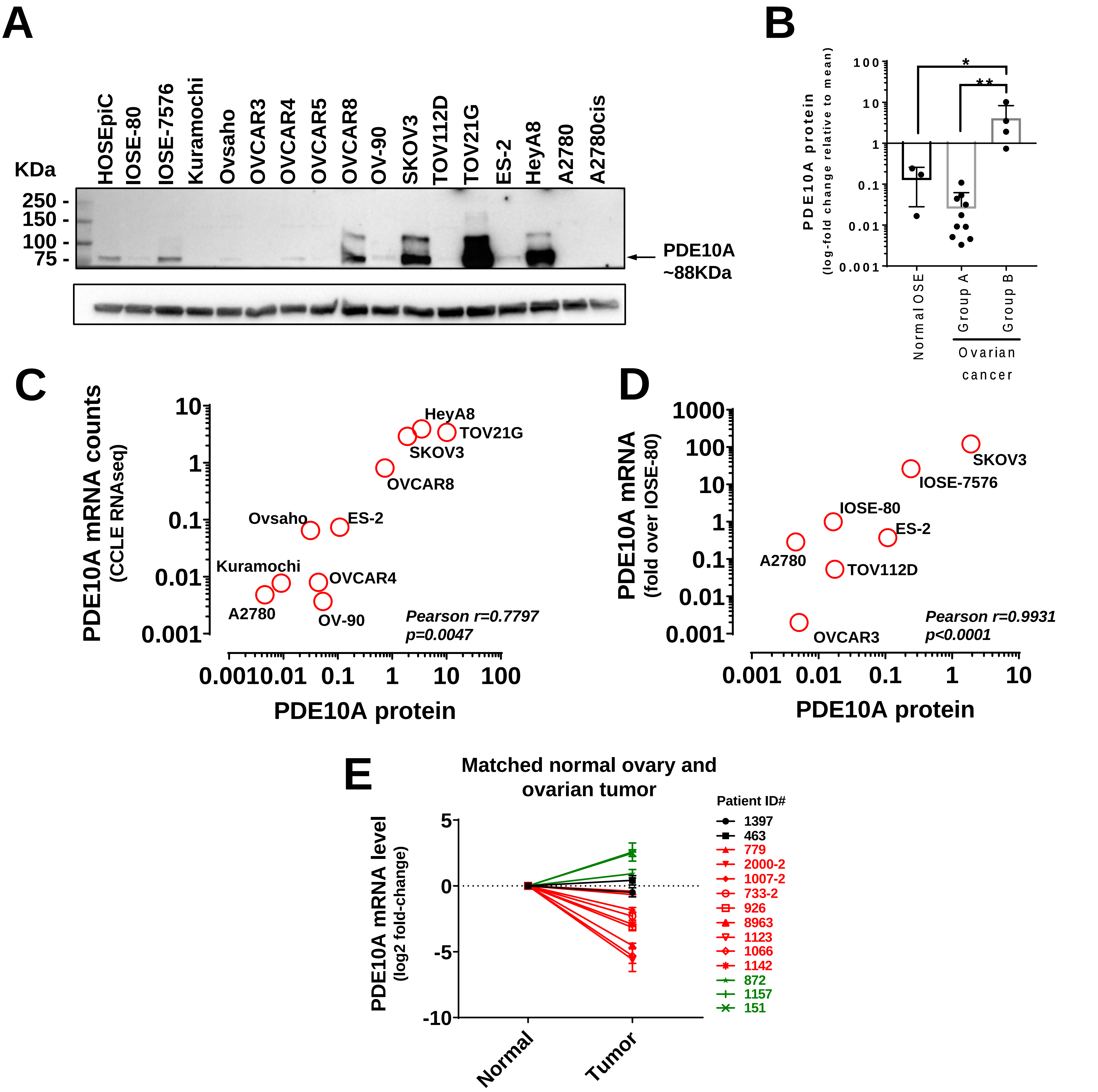
A
KDa
HOSEpiC
IOSE-80
IOSE-7576
Kuramochi
Ovsaho
OVCAR3
OVCAR4
OVCAR5
OVCAR8
OV-90
SKOV3
TOV112D
TOV21G
ES-2
HeyA8
A2780
A2780cis
250 -
150 -
100 -
75 -
PDE10A
~88KDa
B
PDE10A protein
(log-fold change relative to mean)
100
10
1
0.1
0.01
0.001
*
**
Normal OSE
Group A
Group B
Ovarian cancer
C
PDE10A mRNA counts
(CCLE RNAseq)
10
1
0.1
0.01
0.001
HeyA8
TOV21G
SKOV3
OVCAR8
Ovsaho
ES-2
Kuramochi
OVCAR4
A2780
OV-90
Pearson r=0.7797
p=0.0047
0.001
0.01
0.1
1
10
100
PDE10A protein
D
PDE10A mRNA
(fold over IOSE-80)
1000
100
10
1
0.1
0.01
0.001
SKOV3
IOSE-7576
IOSE-80
ES-2
A2780
TOV112D
OVCAR3
Pearson r=0.9931
p<0.0001
0.001
0.01
0.1
1
10
PDE10A protein
E
Matched normal ovary and ovarian tumor
PDE10A mRNA level
(log2 fold-change)
5
0
-5
-10
Normal
Tumor
Patient ID#
1397
463
779
2000-2
1007-2
733-2
926
8963
1123
1066
1142
872
1157
151

Fig S4

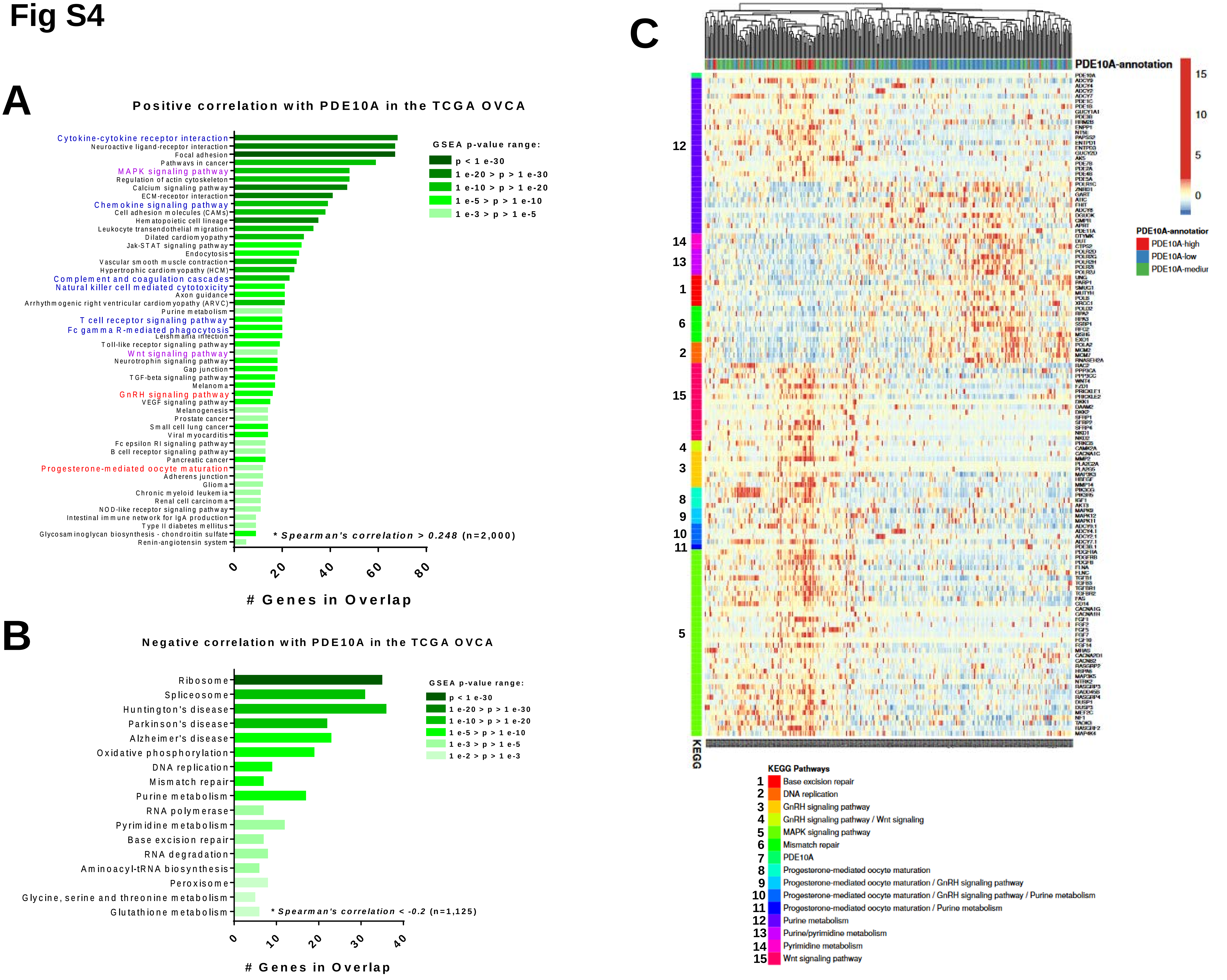

A
Positive correlation with PDE10A in the TCGA OVCA
Cytokine-cytokine receptor interaction
Neuroactive ligand-receptor interaction
Focal adhesion
Pathways in cancer
MAPK signaling pathway
Regulation of actin cytoskeleton
Calcium signaling pathway
ECM-receptor interaction
Chemokine signaling pathway
Cell adhesion molecules (CAMs)
Hematopoietic cell lineage
Leukocyte transendothelial migration
Dilated cardiomyopathy
Jak-STAT signaling pathway
Endocytosis
Vascular smooth muscle contraction
Hypertrophic cardiomyopathy (HCM)
Complement and coagulation cascades
Natural killer cell mediated cytotoxicity
Axon guidance
Arrhythmogenic right ventricular cardiomyopathy (ARVC)
Purine metabolism
T cell receptor signaling pathway
Fc gamma R-mediated phagocytosis
Leishmania infection
Toll-like receptor signaling pathway
Wnt signaling pathway
Neurotrophin signaling pathway
Gap junction
TGF-beta signaling pathway
Melanoma
GnRH signaling pathway
VEGF signaling pathway
Melanogenesis
Prostate cancer
Small cell lung cancer
Viral myocarditis
Fc epsilon RI signaling pathway
B cell receptor signaling pathway
Pancreatic cancer
Progesterone-mediated oocyte maturation
Adherens junction
Glioma
Chronic myeloid leukemia
Renal cell carcinoma
NOD-like receptor signaling pathway
Intestinal immune network for IgA production
Type II diabetes mellitus
Glycosaminoglycan biosynthesis - chondroitin sulfate
Renin-angiotensin system
GSEA p-value range:
p < 1 e-30
1 e-20 > p > 1 e-30
1 e-10 > p > 1 e-20
1 e-5 > p > 1 e-10
1 e-3 > p > 1 e-5
* Spearman's correlation > 0.248 (n=2,000)
0
20
40
60
80
Genes in Overlap
B
Negative correlation with PDE10A in the TCGA OVCA
Ribosome
Spliceosome
Huntington's disease
Parkinson's disease
Alzheimer's disease
Oxidative phosphorylation
DNA replication
Mismatch repair
Purine metabolism
RNA polymerase
Pyrimidine metabolism
Base excision repair
RNA degradation
Aminoacyl-tRNA biosynthesis
Peroxisome
Glycine, serine and threonine metabolism
Glutathione metabolism
GSEA p-value range:
p < 1 e-30
1 e-20 > p > 1 e-30
1 e-10 > p > 1 e-20
1 e-5 > p > 1 e-10
1 e-3 > p > 1 e-5
1 e-2 > p > 1 e-3
* Spearman's correlation < -0.2 (n=1,125)
0
10
20
30
40
Genes in Overlap
C
PDE10A-annotation
15
10
5
0
PDE10A-annotatior
PDE10A-high
PDE10A-low
PDE10A-mediur
12
14
13
1
6
2
15
4
3
8
9
10
11
5
KEGG
KEGG Pathways
1 Base excision repair
2 DNA replication
3 GnRH signaling pathway
4 GnRH signaling pathway / Wnt signaling
5 MAPK signaling pathway
6 Mismatch repair
7 PDE10A
8 Progesterone-mediated oocyte maturation
9 Progesterone-mediated oocyte maturation / GnRH signaling pathway
10 Progesterone-mediated oocyte maturation / GnRH signaling pathway / Purine metabolism
11 Progesterone-mediated oocyte maturation / Purine metabolism
12 Purine metabolism
13 Purine/pyrimidine metabolism
14 Pyrimidine metabolism
15 Wnt signaling pathway

Fig S5

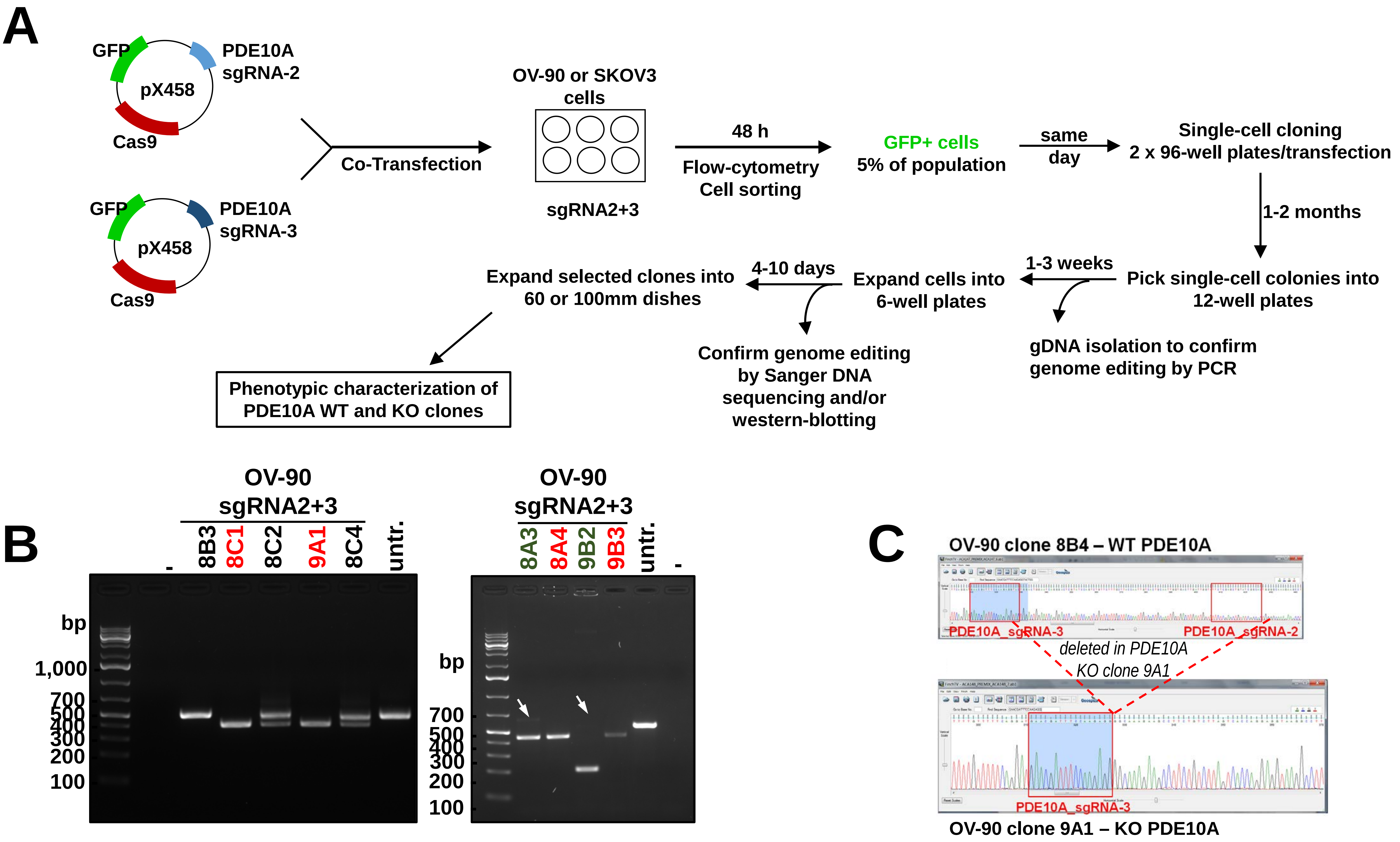
A
GFP
PDE10A sgRNA-2
pX458
Cas9
GFP
PDE10A sgRNA-3
pX458
Cas9
Co-Transfection
OV-90 or SKOV3 cells
sgRNA2+3
48 h
Flow-cytometry
Cell sorting
GFP+ cells
5% of population
same day
Single-cell cloning
2 x 96-well plates/transfection
1-2 months
Pick single-cell colonies into 12-well plates
1-3 weeks
gDNA isolation to confirm genome editing by PCR
Expand cells into 6-well plates
4-10 days
Confirm genome editing by Sanger DNA sequencing and/or western-blotting
Expand selected clones into 60 or 100mm dishes
Phenotypic characterization of PDE10A WT and KO clones
B
OV-90
sgRNA2+3
- 8B3 8C1 8C2 9A1 8C4 untr.
bp
1,000
700
500
400
300
200
100
OV-90
sgRNA2+3
8A3 8A4 9B2 9B3 untr. -
bp
700
500
400
300
200
100
C
OV-90 clone 8B4 – WT PDE10A
PDE10A_sgRNA-3
PDE10A_sgRNA-2
deleted in PDE10A KO clone 9A1
PDE10A_sgRNA-3
OV-90 clone 9A1 – KO PDE10A

Fig S6

A

Selected Overlapping Genes:

- ADAMSTS12
- CBFA2T3
- COL1A2
- COL6A1
- CYP1B1
- DVL2
- EDNRA
- ESR1
- FZD1
- GLI1
- HHIP
- PDE3B

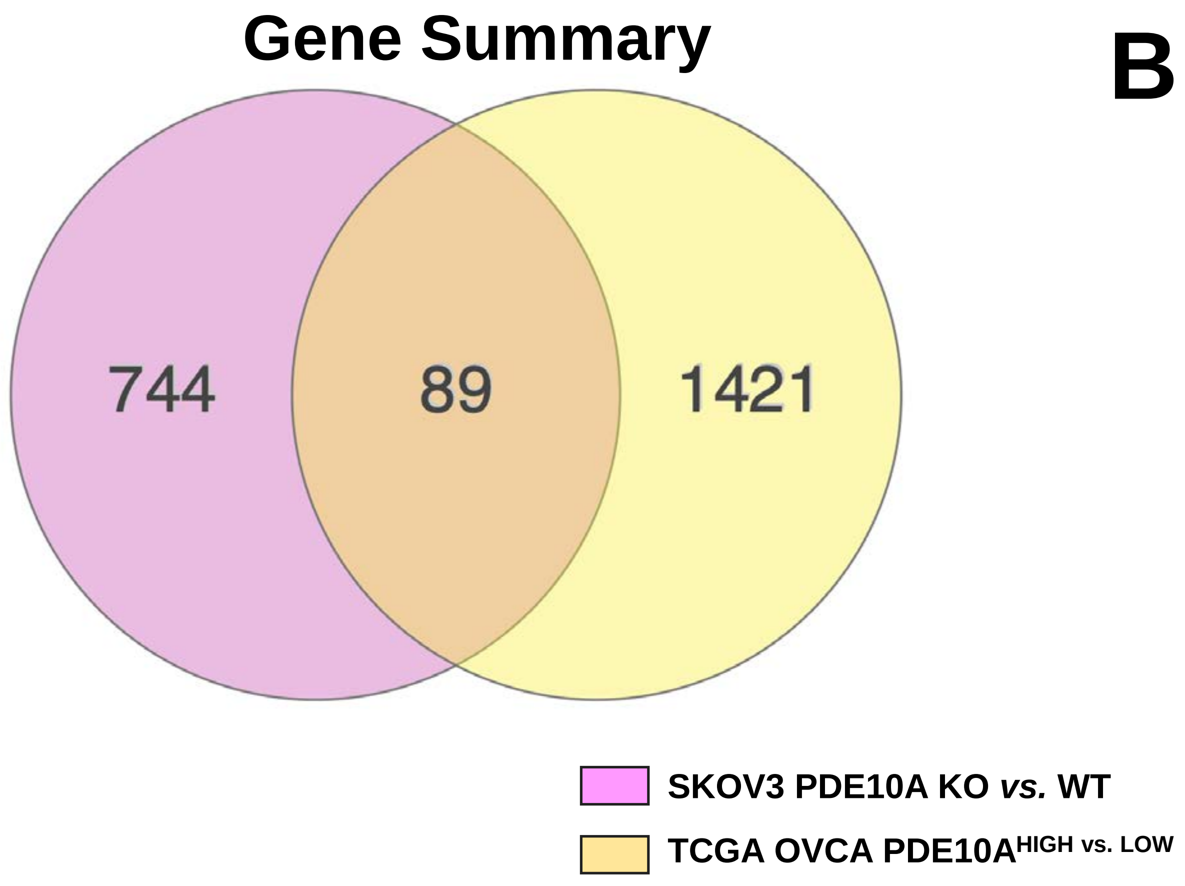

B

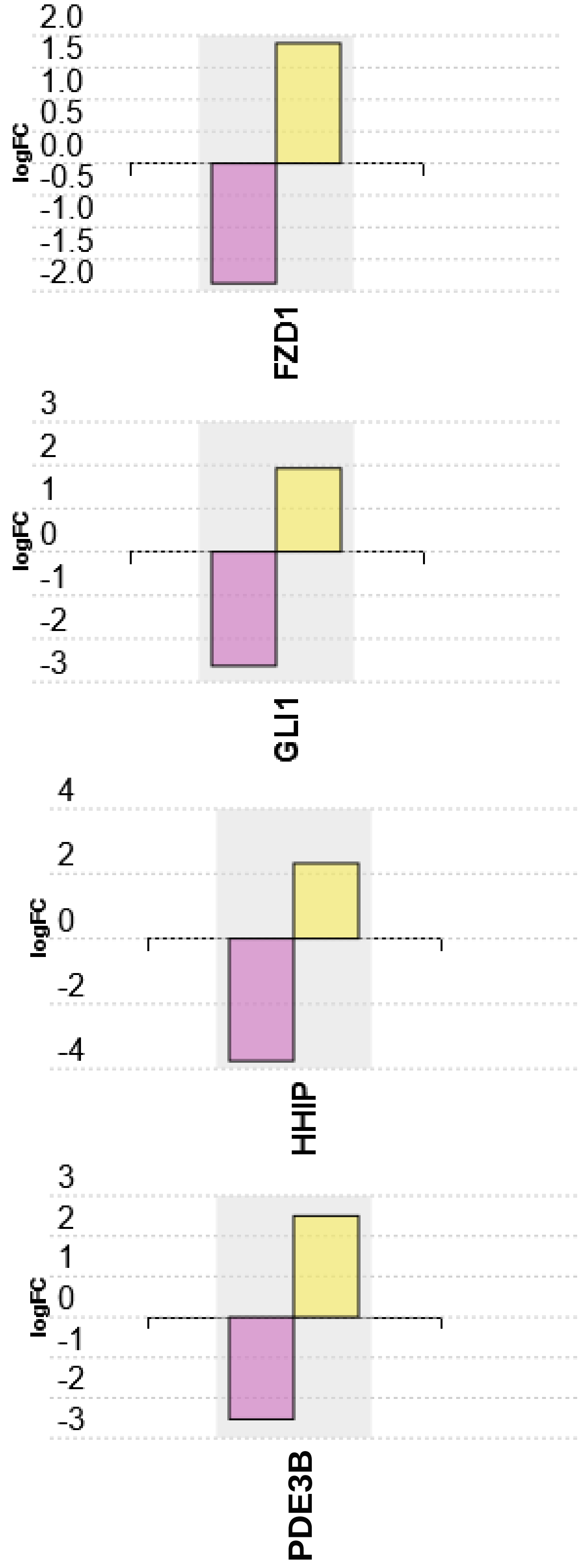

C

Overlapping Pathways:

- Alzheimer disease
- Breast cancer
- Calcium signaling pathway
- cAMP signaling pathway
- Cell adhesion molecules
- Cholinergic synapse
- Dilated cardiomyopathy
- Gastric cancer
- Human papillomavirus infection
- Malaria
- Pathways in cancer
- Proteoglycans in cancer
- Regulation of lipolysis in adipocytes

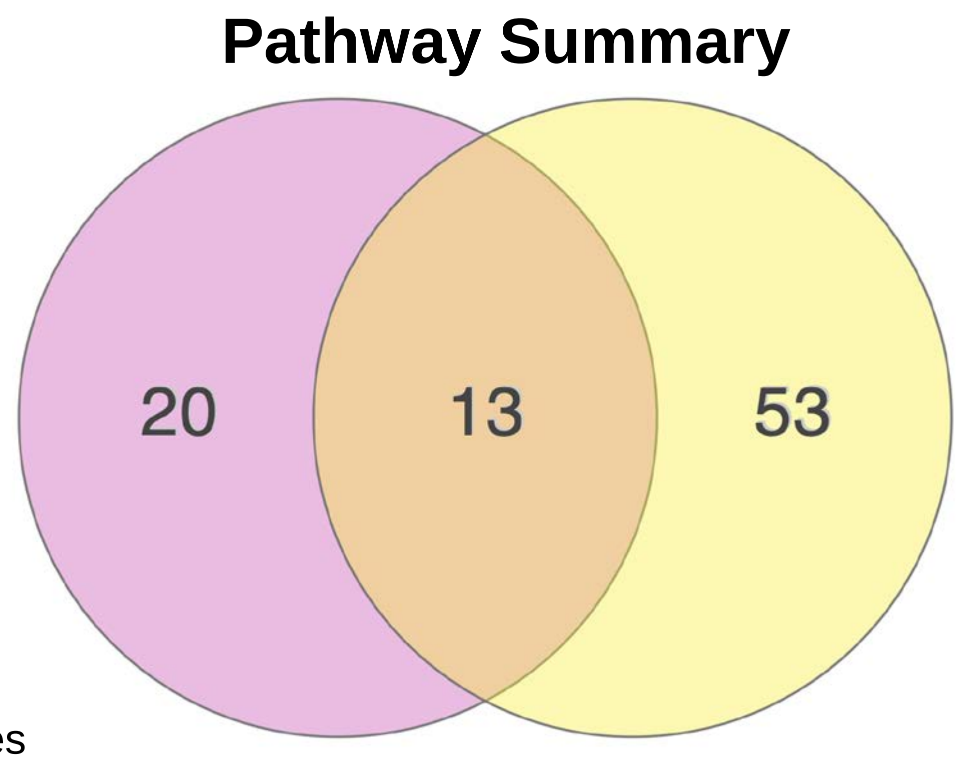

Fig S7A

TCGA OVCA PDE10A^{HIGH} *vs.* PDE10A^{LOW}
Pathways in Cancer: Log-fold changes

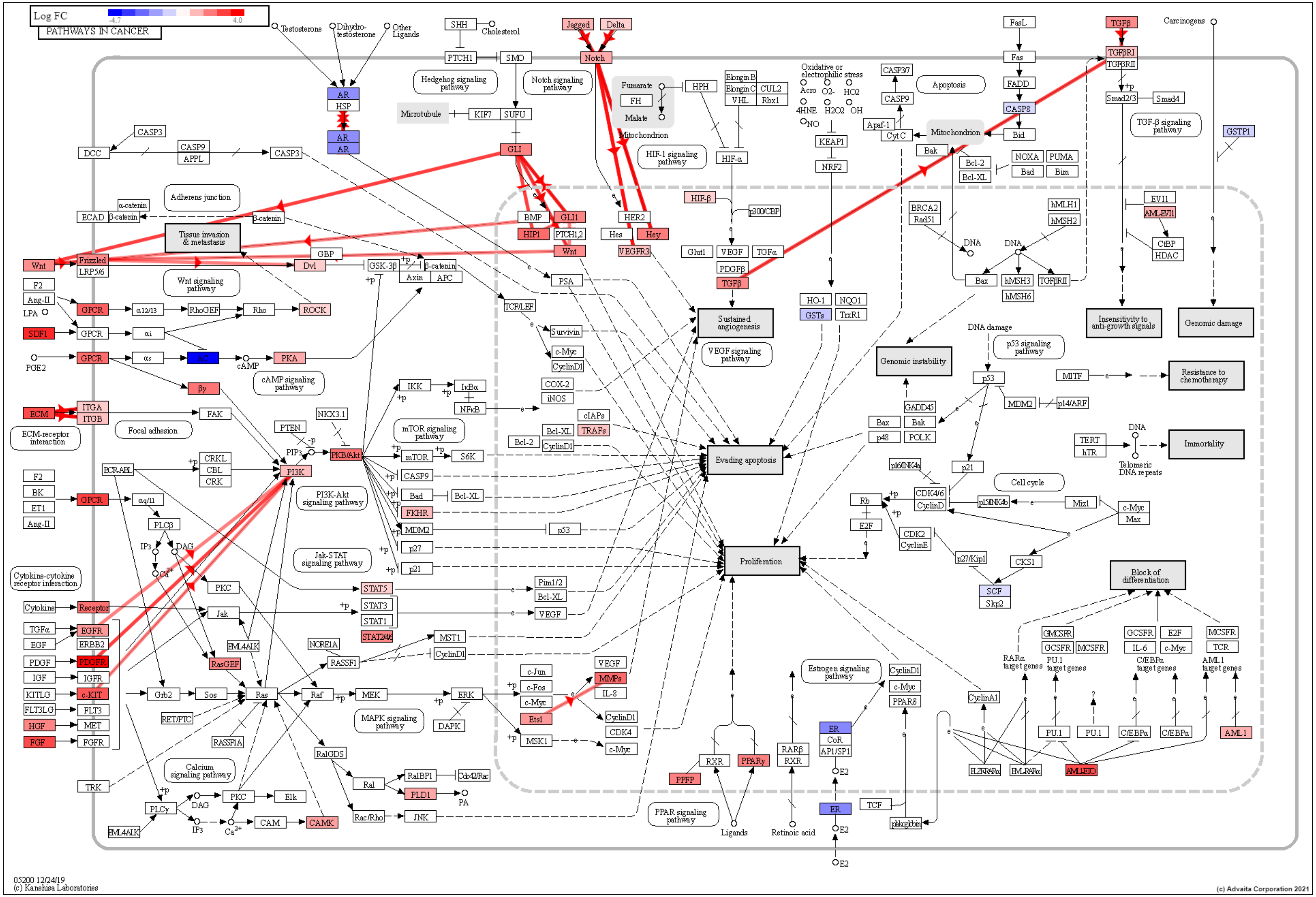

Fig S7B

SKOV3 PDE10A WT *vs.* KO
Pathways in Cancer: Log-fold changes

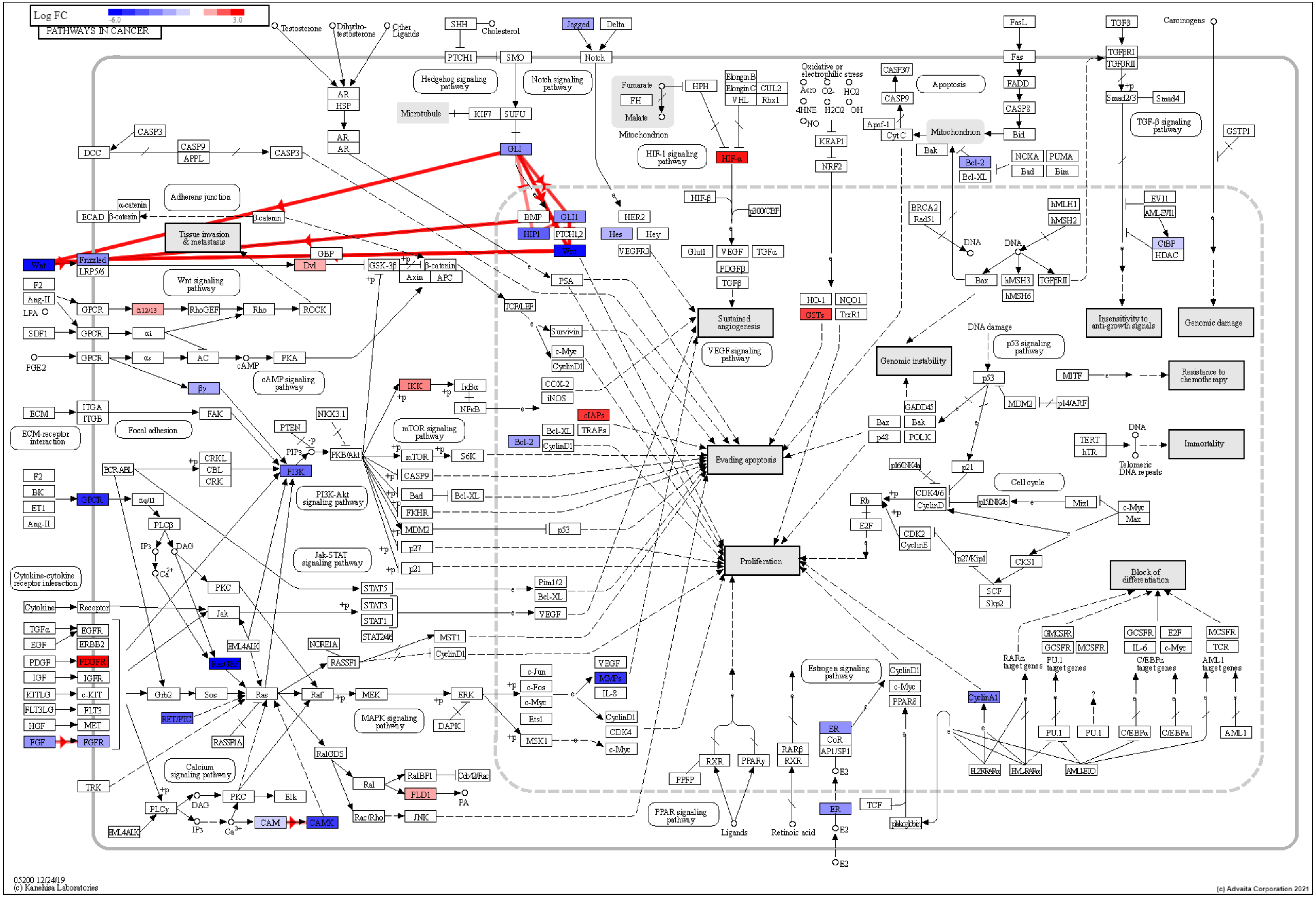

Fig S7C

TCGA OVCA PDE10A^{HIGH} *vs.* PDE10A^{LOW}
Pathways in Cancer: Perturbation

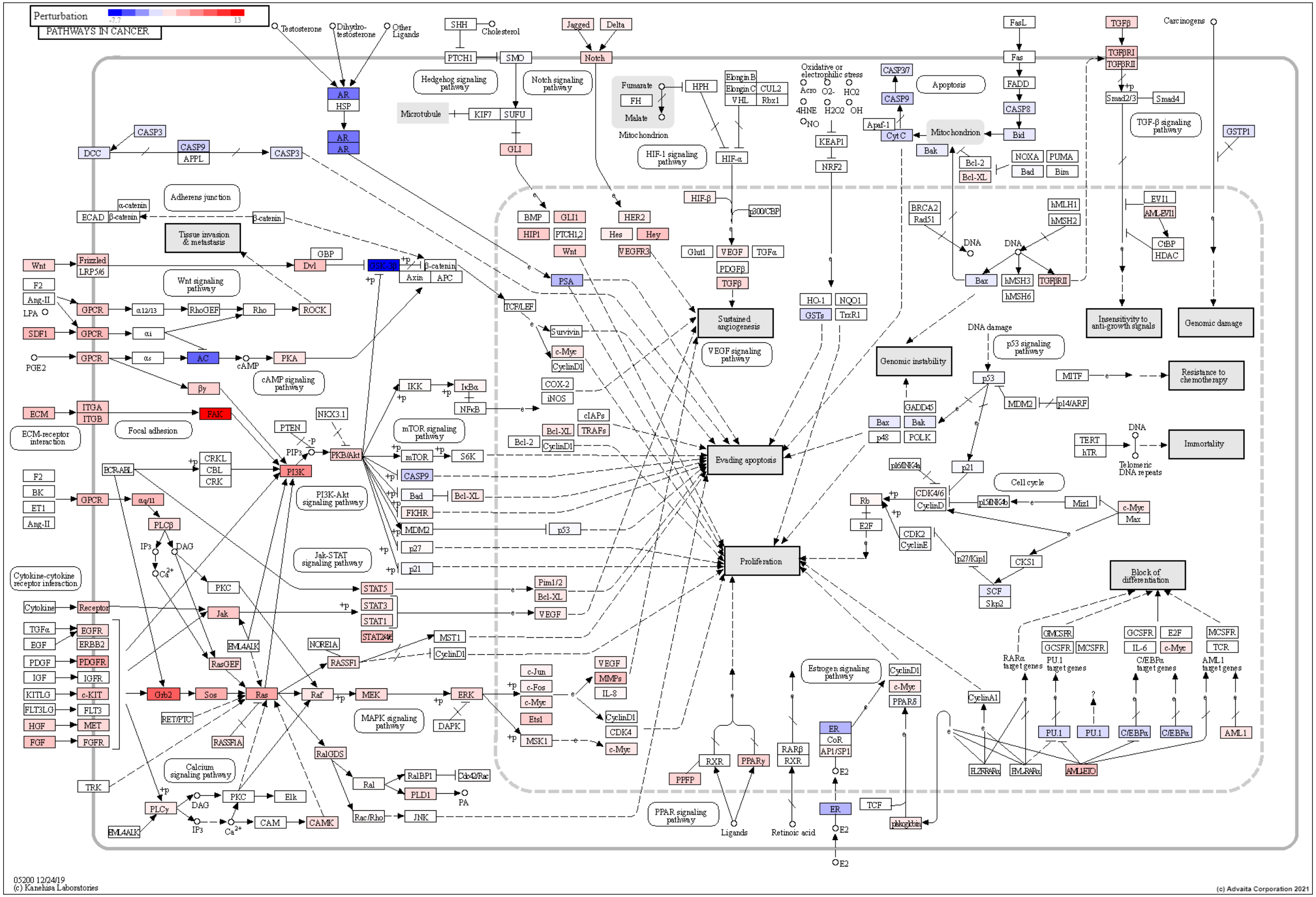

Fig S7D

SKOV3 PDE10A WT *vs.* KO
Pathways in Cancer: Perturbation

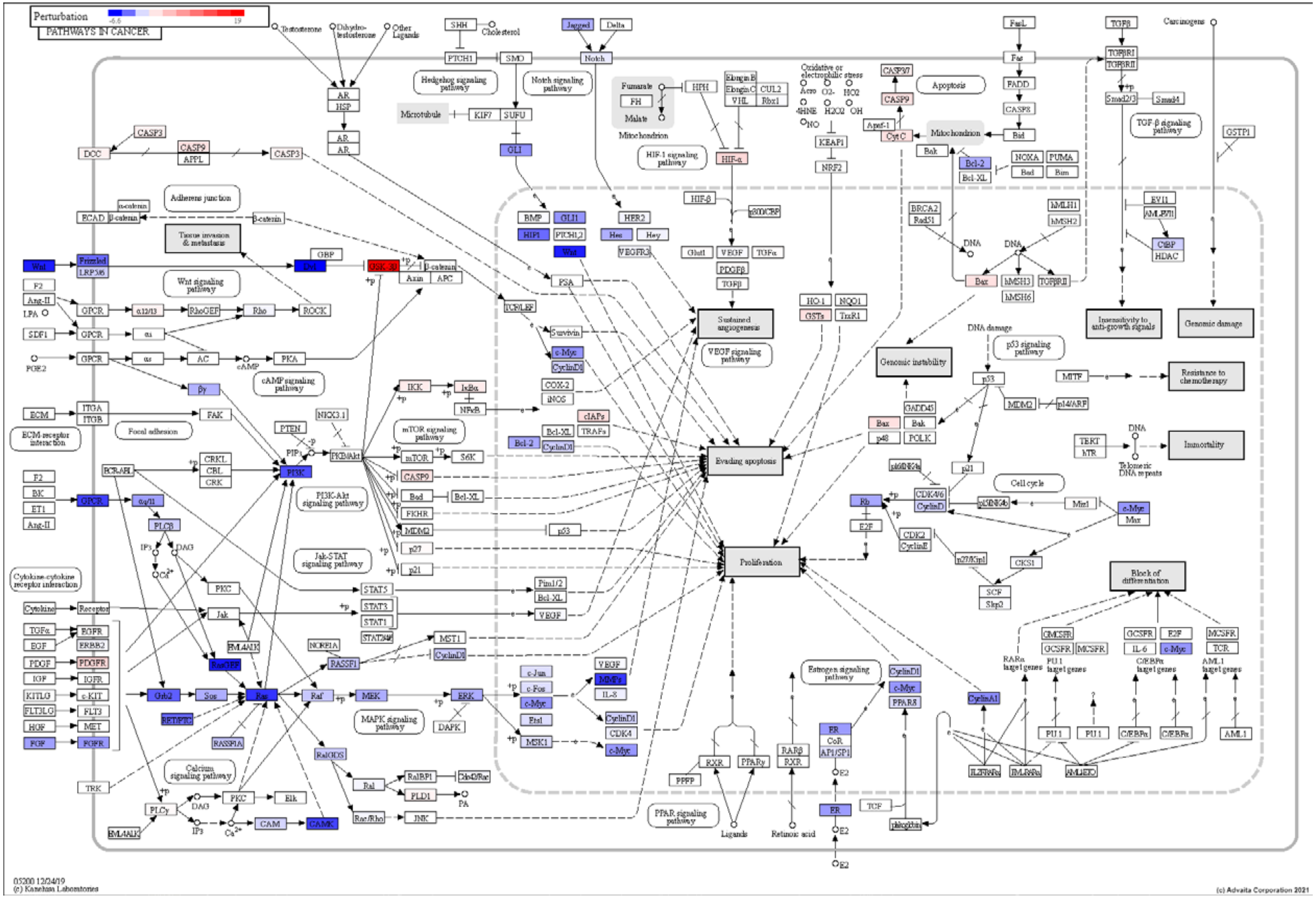

Fig S7E

TCGA OVCA PDE10A^{HIGH} *vs.* PDE10A^{LOW}
Breast Cancer Pathway: Log-fold changes

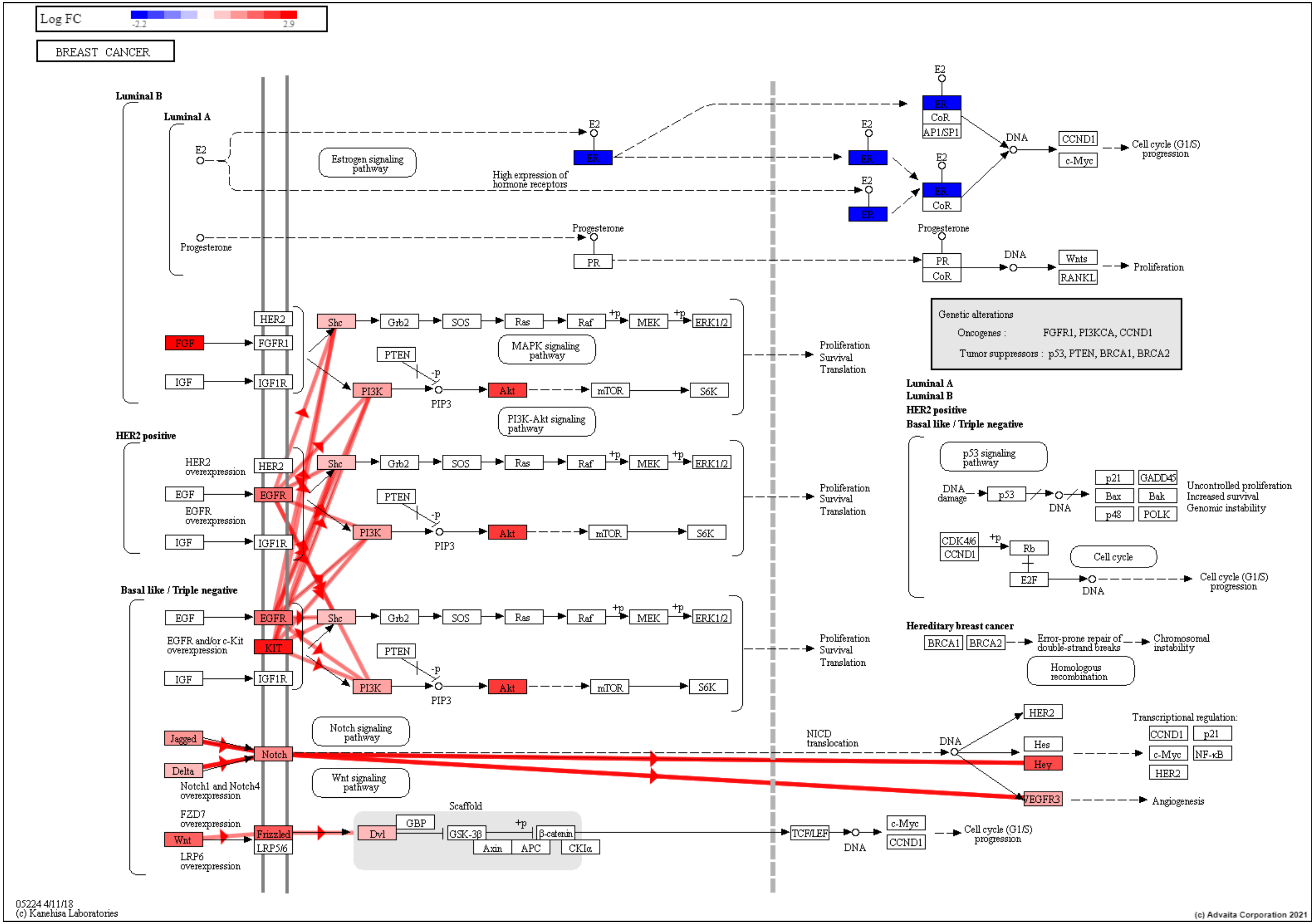

Fig S7F

SKOV3 PDE10A WT *vs.* KO
Breast Cancer Pathway: Log-fold changes

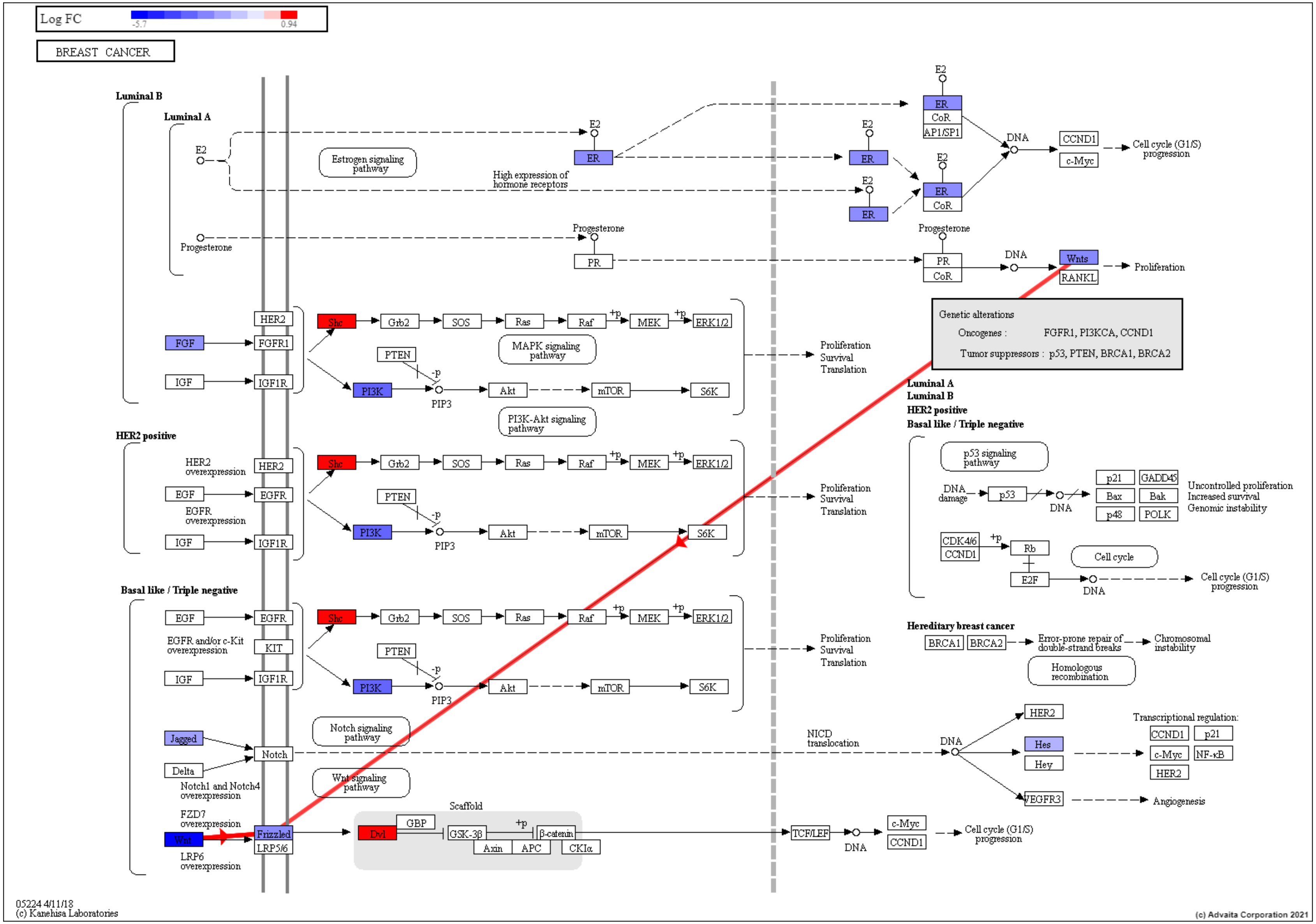

Fig S7G

TCGA OVCA PDE10A^{HIGH} *vs.* PDE10A^{LOW} Breast Cancer Pathway: Perturbation

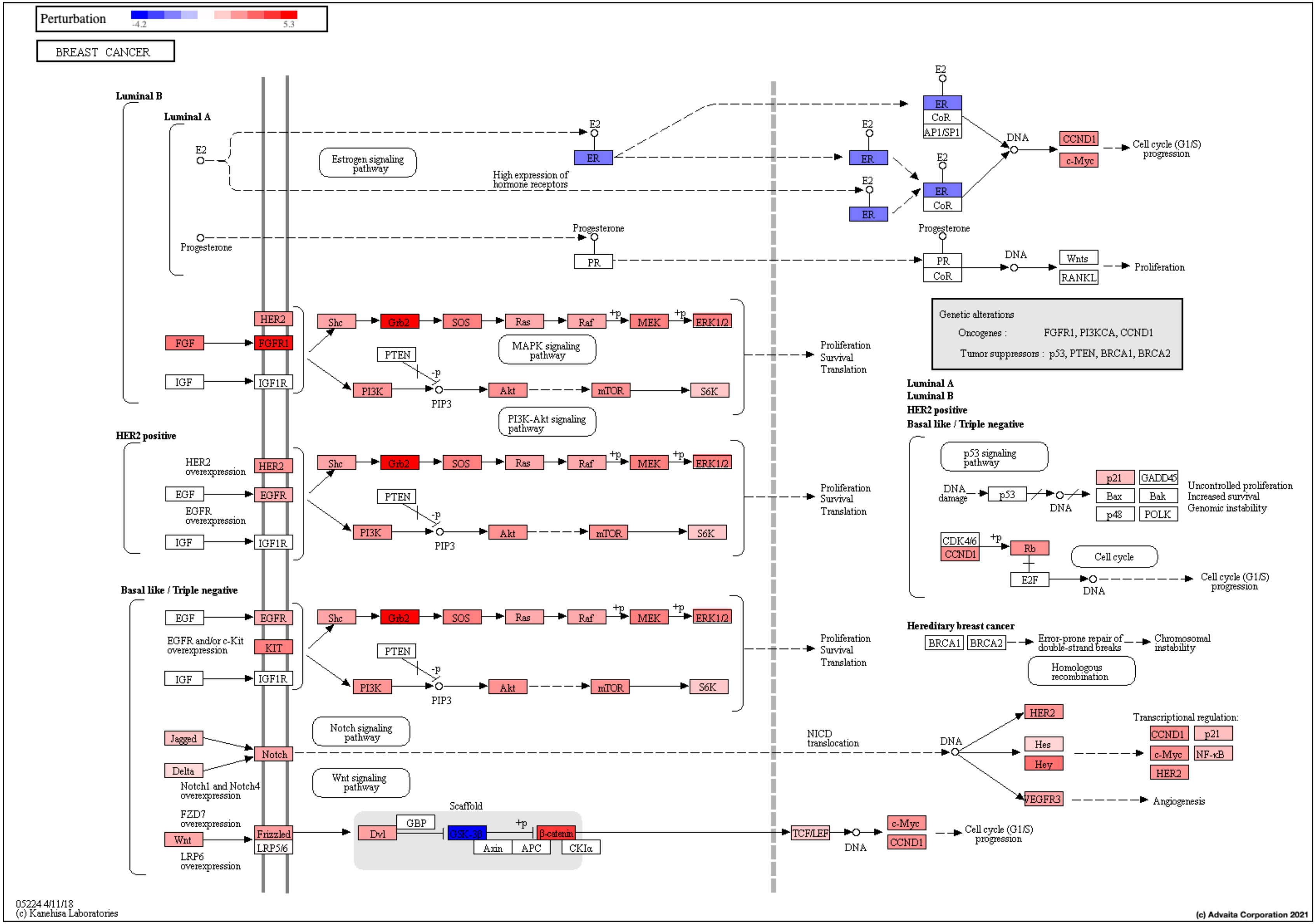

Fig S7H

SKOV3 PDE10A WT *vs.* KO
Breast Cancer Pathway: Perturbation

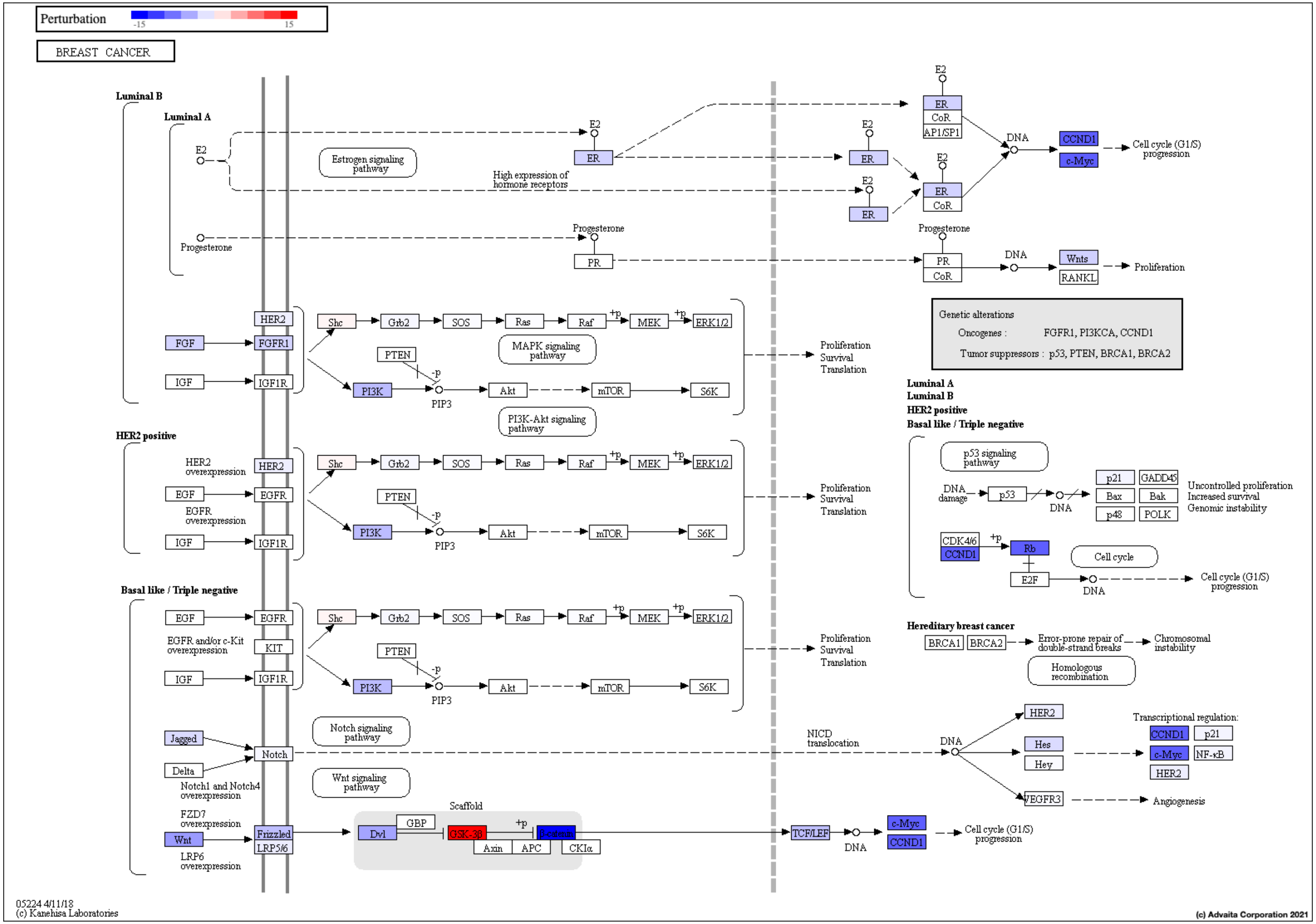

Fig S8

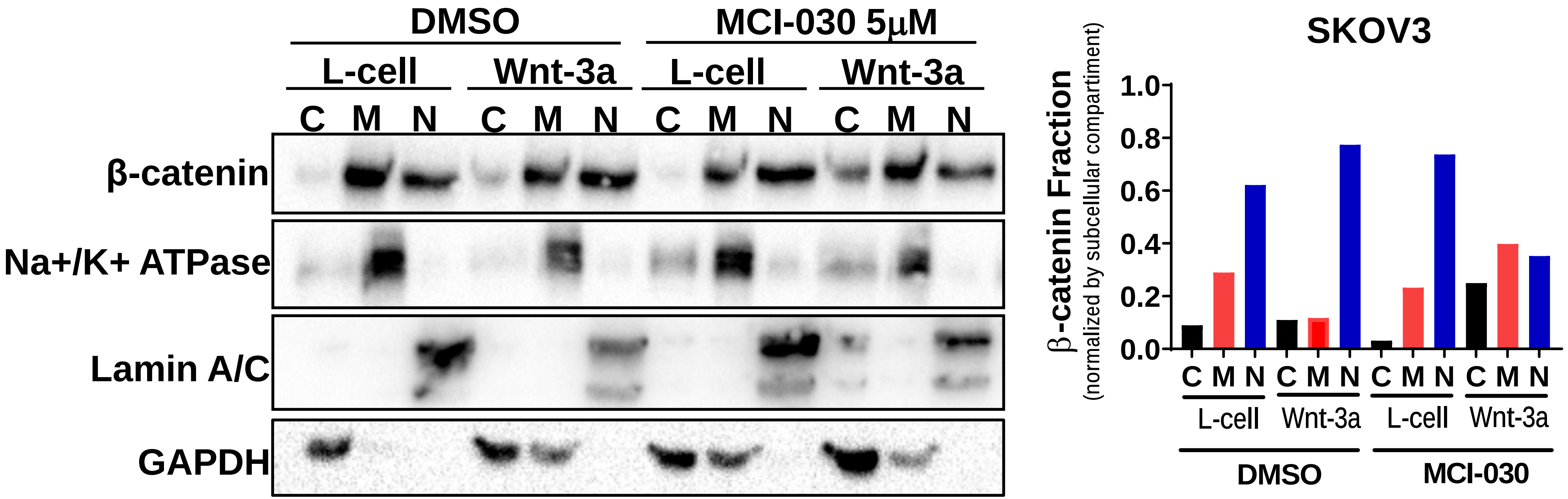
DMSO
MCI-030 5µM
L-cell
Wnt-3a
L-cell
Wnt-3a
C M N C M N C M N C M N
β-catenin
Na+/K+ ATPase
Lamin A/C
GAPDH
SKOV3
β-catenin Fraction
(normalized by subcellular compartiment)
1.0
0.8
0.6
0.4
0.2
0.0
C M N C M N C M N C M N
L-cell
Wnt-3a
L-cell
Wnt-3a
DMSO
MCI-030
