# Supplementary material for: Phosphodiesterase 10A (PDE10A) as a novel target to suppress β-catenin and RAS signaling in epithelial ovarian cancer
Source: J Ovarian Res. 2022 Nov 2;15:120. doi: 10.1186/s13048-022-01050-9 (PMC9632086; doi:10.1186/s13048-022-01050-9)
Supplement: Supplementary file 1 — Additional file 1. [file 13048_2022_1050_MOESM1_ESM.zip › Supplemental Figure Legends.docx]

**Figure Legends for Supplemental Figures**

**Figure S1: PDE10A DNA copy number variation is associated with PDE10A mRNA expression levels and survival in ovarian cancer patients. (A)** PDE10A DNA copy number distribution in the TCGA ovarian tumor samples. **(B)** PDE10A DNA copy number correlates with mRNA expression in ovarian tumors. Error bars, SD; ** p<0.01 (ordinary one-way ANOVA). **(C-D)** Kaplan-Meyer overall (C) and disease-free (D) survival analysis of TCGA ovarian cancer patients stratified by PDE10A DNA copy number variation. All data was obtained from the cBioportal.

**Figure S2: PDE10A high mRNA levels correlate with poor prognosis in ovarian cancer.** **(A)** Kaplan-Meyer disease free survival analysis of TCGA ovarian cystadenocarcinoma patients stratified by PDE10A mRNA expression levels. PDE10A^high^ and PDE10A^low^ correspond to mRNA expression 2x higher (n=10) and 2x lower (n=15) than mean PDE10A mRNA levels in the population, respectively. **(B)** Kaplan-Meyer overall survival analysis of patients from Duke study for ovarian cancer stratified by PDE10A mRNA expression levels (PDE10A median used for stratification of high vs. low). Data extracted from Prognoscan.

**Figure S3: PDE10A mRNA levels in ovarian cancer cell lines and matched normal ovaries and ovary tumors. (A)** Uncropped image for western-blotting with Abcam rabbit monoclonal PDE10A antibody. **(B)** Densitometry quantitation of western-blotting showing PDE10A protein (normalized to GAPDH as loading control) levels in ovarian surface epithelial cell lines (OSE: HOSEpiC, IOSE-80 and IOSE-7576), compared to two groups of ovarian cancer cell lines: Group A (Kuramochi, Ovsaho, OVCAR3, OVCAR4, OVCAR5,OV-90, TOV112D, ES-2, A2780, A2780cis), and Group B (OVCAR8, SKOV3, TOV21G and HeyA8). Error bars, SD; * p<0.05 and ** p<0.01 (ordinary one-way ANOVA). **(C-D)** Correlation of PDE10A protein quantified by western-blotting with PDE10A mRNA counts in the CCLE database (C) or with PDE10A mRNA measured by qRT-PCR in our “in house” cell lines (D). Relative expression was calculated with the ΔΔCt method using GPS1 as housekeeping gene. Y-axis represents fold-change relative to IOSE-80. CCLE PDE10A mRNA counts were retrieved from the cBioportal. **(E)** PDE10A mRNA levels measured by qRT-PCR in ovary tumor and normal ovary clinical specimens deposited at our institutional Biobank. Relative expression was calculated with the ΔΔCt method using GPS1 as housekeeping gene. Y-axis represents log2 fold-change of each ovary tumor to its respective matched normal ovary tissue.

**Figure S4: Pathways correlated with PDE10A expression in the TCGA ovarian tumors. (A)** KEGG pathway analysis of top 2,000 genes with mRNA expression levels positively correlated with PDE10A mRNA in ovarian tumors from the TCGA (Spearman r > 0.248; q-value < 0.0001). **(B)** KEGG pathway analysis of top 1,125 genes with mRNA expression levels negatively correlated with PDE10A mRNA in ovarian tumors from the TCGA (Spearman r < -0.2; q-value < 0.0025). **(C)** Heatmap showing selected KEGG pathways correlated with PDE10A in ovarian tumors.

**Figure S5: Screening and validation of OV-90 PDE10A knockout cells. (A)** Flow-chart of CRISPR-Cas9 strategy used to knockout PDE10A in OV-90 cells with co-transfection of two sgRNAs that target exon 7 of PDE10A to delete a predicted 97bp segment that disrupts the ORF. (B) Representative PCR results for screening of clones using primers gRNA2-7i-fw3 and gRNA2-7i-rv3 as described in Supplemental Methods and Table S4. (C) Sanger sequencing traces comparing the PCR fragment amplified as described in (B) for PDE10A WT clone 8B4 and PDE10A KO clone 9A1 showing the deletion at the sgRNA sites as predicted.

**Figure S6: Pathway and gene comparisons between TCGA and PDE10A KO cells RNAseq.** A meta-analysis was used in the iPathway Guide impact analysis to compare the TCGA and PDE10A KO cell RNAseq datasets (as described in Fig1 and 2). **(A)** Venn diagram depicting the number of genes significantly impacted in both datasets and overlapping genes, with selected overlapping genes listed. **(B)** Boxplots with fold-changes for 4 genes of interest: FZD1, GLI1, HHIP, and PDE3B. **(C)** Venn diagram depicting the number of pathways significantly impacted in both datasets and overlapping pathways, with all overlapping pathways listed.

**Figure S7: Comparison of cancer-related pathway gene expression between TCGA and PDE10A KO cells RNAseq.** Pathway diagrams for “pathways in cancer” (A-D) and “breast cancer” (E-H) comparing Log2-fold change and perturbation analyses for both TCGA and PDE10A KO cells RNAseq (as described in Figures 1 and 2).

**Figure S8:** **Effect of Pf-2545920 upon Wnt-3a-induced β-catenin nuclear translocation in SKOV3 cells.** Western-blotting detection of β-catenin in cytoplasmic [C], membrane [M], and nuclear [N] compartments obtained by subcellular fractionation of SKOV3 cells pre-incubated with MCI-030 or DMSO for 2 hours followed by stimulation with Wnt-3A or L-cell media for additional 5 h. Loading controls were Na+/K+ATPase for membranous, Lamin A/C for nuclear, and GAPDH for cytoplasmic fractions. Quantitation of β-catenin fraction was calculated by measuring β-catenin fraction for each compartment and normalizing by their respective loading control markers.
