# Supplementary material for: Phosphodiesterase 10A (PDE10A) as a novel target to suppress β-catenin and RAS signaling in epithelial ovarian cancer
Source: J Ovarian Res. 2022 Nov 2;15:120. doi: 10.1186/s13048-022-01050-9 (PMC9632086; doi:10.1186/s13048-022-01050-9)
Supplement: Supplementary file 1 — Additional file 1. [file 13048_2022_1050_MOESM1_ESM.zip › Supplemental Tables S1_S2_S3_S4_S26.pdf]

Table S1: Antibodies used in the study

Antibody	Manufacturer	Catalog #	Dilution
**Western Blotting**			
PDE10A	Abcam	Ab227829	1 : 1,000
GAPDH	Cell Signaling	2118S	1 : 1,000
Non-Phospho (active) β-Catenin	Cell Signaling	8814S	1 : 1,000
β-Catenin	Cell Signaling	8480S	1 : 1,000
Phospho-VASP (ser157)	Cell Signaling	3111S	1 : 250
Phospho-VASP (ser239)	Cell Signaling	3114S	1 : 250
VASP	Cell Signaling	3132S	1 : 250
Survivin	Cell Signaling	2808S	1 : 1,000
Cyclin D	Cell Signaling	2978S	1 : 1,000
c-MYC	Cell Signaling	5605S	1 : 1,000
Cleaved PARP	Cell Signaling	5625S	1 : 1,000
PARP	Cell Signaling	9532S	1 : 1,000
Phospho-MAPK (ERK1/2) (Thr202/Tyr204)	Cell Signaling	9101S	1 : 1,000
ERK	Cell Signaling	4695S	1 : 1,000
Phospho-AKT (Thr308)	Cell Signaling	13038S	1 : 1,000
Phospho-AKT (Ser473)	Cell Signaling	4060S	1 : 1,000
AKT	Cell Signaling	4691S	1 : 1,000
Lamin A/C	Cell Signaling	4777S	1 : 1,000
Na+/K+ATPase	Cell Signaling	3010S	1 : 1,000
Anti-rabbit IgG, HRP-linked antibody	Cell Signaling	704P2	1 : 5,000
Anti-mouse IgG, HRP-linked Antibody	Cell Signaling	7076S	1 : 3,000
**Immunofluorescence assay for confocal imaging**			
β-Catenin	Cell Signaling	8480S	1 : 200
Goat Anti-Rabbit IgG H&L (Alexa Fluor® 488)	Abcam	ab150077	1 : 1,000
DAPI	ThermoFisher Scientific	D1306	300 nM
**Immunoprecipitation for PDE10A activity assay**			
PDE10A	Santa Cruz Biotech	SC-515023	
normal mouse IgG	Jackson ImmunoResearch	015-000-003	

Table S2: Cell lines used in the study

Cell Line	Source	Culture Medium	Year of acquisition
**HOSEpiC**	ScienceCell Research	Ovarian Epithelial Cell Medium (ScienceCell Research)	2014
**IOSE-7576**	OVCARE, British Columbia	1:1 M199 : MCDB105 + 5% FBS + pen/strep	2014
**IOSE-80**	OVCARE, British Columbia	1:1 M199 : MCDB105 + 5% FBS + pen/strep	2014
**ES-2**	ATCC	McCoys 5A + 10% FBS + pen/strep	2014
**OV-90**	ATCC	1:1 M199 : MCDB105 + 15% FBS + pen/strep	2014
**SKOV3**	ATCC	McCoys 5A + 10% FBS + pen/strep	2014
**TOV112D**	ATCC	1:1 M199 : MCDB105 + 15% FBS + pen/strep	2014
**TOV21G**	ATCC	1:1 M199 : MCDB105 + 15% FBS + pen/strep	2014
**OVCAR-3**	NCI Frederick Cancer DCT Tumor Repository	RPMI + 20% FBS + 0.01mg/ml insulin + pen/strep	2014
**OVCAR-4**	NCI Frederick Cancer DCT Tumor Repository	RPMI1640 +10%FBS +2mM L-glutamine +pen/strep	2016
**OVCAR-5**	NCI Frederick Cancer DCT Tumor Repository	RPMI1640 +10%FBS +2mM L-glutamine +pen/strep	2016
**OVCAR-8**	NCI Frederick Cancer DCT Tumor Repository	RPMI1640 +10%FBS +2mM L-glutamine +pen/strep	2016
**HeyA8**	MD Anderson Cancer Center	RPMI1640 +10%FBS +2mM L-glutamine +pen/strep	2015
**Kuramochi**	Japanese Collection of Research Bioresources Cell Bank	RPMI1640 +10%FBS +pen/strep	2016
**Ovsaho**	Japanese Collection of Research Bioresources Cell Bank	RPMI1640 +10%FBS +pen/strep	2016
**A2780**	Sigma Aldrich	RPMI-1640 + 2mM Glutamine + 10% FBS + pen/strep	2019
**A2780cis**	Sigma Aldrich	RPMI-1640 + 2mM Glutamine + 10% FBS + pen/strep	2019

*P/S: penicillin/streptomycin

Table S3: sgRNA oligonucleotides, plasmids and primers used to generate PDE10A KO cell lines using CRISPR/Cas9 technology

**sgRNA oligonucleotides synthesized to be inserted into**		
**sgRNA oligonucleotides**	**Sequence (5’ → 3’)**	**Modification**
PDE10A-gRNA2-top	CACCGTTCTCGAGCTGTATCGGCAC	phosphorylated
PDE10A-gRNA2-bottom	AAACGTGCCGATACAGCTCGAGAAC	phosphorylated
PDE10A-gRNA3-top	CACCGGAACGATTTCCAAGAGGTAC	phosphorylated
PDE10A-gRNA3-bottom	AAACGTACCTCTTGGAAATCGTTCC	phosphorylated

**Primers to confirm sgRNA targeted sequence at PDE10A genomic locus by PCR**	
**Primer Name**	**Sequence (5’ → 3’)**
gRNA2-7i-fw3	AAGACTGTTCCGGCACATAG
gRNA2-7i-rv3	CACCTTCAATGAGCTAGGTACAAC

Table S4: Sequences for primers for Real-time quantitative PCR

Primers for RT-qPCR		
**Primer Name**	**Sequence (5’ → 3’)**	**Reference**
PDE10A-fw3	CCGTCTTTTGTGCTTTAGCC	
PDE10A-rv3	CAGCTTTTCCATCGTTACCC	
GPS1-fw	AAGATGCTGGACGAGATGAAGGA	*
GPS1-rv	ACGGTTGCGAATCTGGGTGTA	*

n/a: not applicable

* Lakics V, Karran EH, Boess FG (2010) Quantitative comparison of phosphodiesterase mRNA distribution in human brain and peripheral tissues Neuropharmacol 59: 367-74. doi: 10.1016/j.neuropharm.2010.05.004.

Table S26: IC_{50} Values for growth inhibition of PDE10A Inhibitors in Ovarian Cell Lines

Pf-2545920:

Cell line:	IC_{50} value:
HOSEpiC	7.6 μM
IOSE-80	12.2 μM
IOSE-7576	20.8 μM
IGR-OV1	20.3 μM
OVCAR3	18.4 μM
OVCAR4	11.4 μM
OVCAR8	17.0 μM
SKOV3	28.6 μM
OV-90	12.7 μM

MCI-030

Cell line:	IC_{50} value:
HOSEpiC	1.61 μM
IOSE-80	0.62 μM
IOSE-7576	0.45 μM
SKOV3	0.53 μM
SKOV3-IP1	0.28 μM
OV-90	0.56 μM
ES-2	0.91 μM
TOV-112D	1.07 μM
A2780	0.15 μM
A2780 CP70	0.50 μM
CAOV3	0.46 μM
OV-90 PDE10A WT 8B3	0.46 μM
OV-90 PDE10A KO 9A1	2.67 μM
OV-90 PDE10A KO 8C1	1.54 μM
OV-90 PDE10A KO 4C4	1.24 μM
OV-90 PDE10A KO 9B2	1.51 μM
SKOV3 PDE10A EV 1B9	0.43 μM
SKOV3 PDE10A KO 2F4	1.99 μM
SKOV3 PDE10A KO 2C10	0.98 μM
SKOV3 PDE10A KO 5H5	1.71 μM
