# Supplementary material for: Phosphodiesterase 10A (PDE10A) as a novel target to suppress β-catenin and RAS signaling in epithelial ovarian cancer
Source: J Ovarian Res. 2022 Nov 2;15:120. doi: 10.1186/s13048-022-01050-9 (PMC9632086; doi:10.1186/s13048-022-01050-9)
Supplement: Supplementary file 1 — Additional file 1. [file 13048_2022_1050_MOESM1_ESM.zip › SupplementalMethods.docx]

**Phosphodiesterase 10A (PDE10A) as a novel target to suppress β-catenin and RAS signaling in ovarian cancer**

Rebecca M. Borneman^1^, Elaine Gavin^1^, Alla Musiyenko^1^, Annelise M. Wilhite^1^, Wito Richter^2^, Kevin J. Lee^3^, Crossman David K^4^, Joel F. Andrews^5^, Steven McClellan^6^, Ileana Aragon^2^, Antonio B. Ward^3^, Xi Chen^3^, Adam B. Keeton^3^, Kristy Berry^3^, Gary A. Piazza^3^, Jennifer M. Scalici^1^, Luciana Madeira da Silva^1,*^

**Generation of OV-90 and SKOV3 PDE10A knockout cell lines using CRISPR/Cas9 genome editing:** We followed the procedure described by Dr. Feng Zhang’s and colleagues (1) to apply the CRISPR/Cas9 technology of genome editing to disrupt the open reading frame (ORF) of human PDE10A in two ovarian cancer cell lines, OV-90 and SKOV3. We used their recommended CRISPR Design Tool to select two sgRNA targeting sequences specifically in the exon 7 of human PDE10A:

**PDE10A sgRNA-2**

____20nt-sgRNA2_____

5' CACCGTTCTCGAGCTGTATCGGCAC 3'

3' CAAGAGCTCGACATAGCCGTGCAAA 5'

PDE10A-gRNA2-top: 5' CACCGTTCTCGAGCTGTATCGGCAC 3'

PDE10A-gRNA2-bottom: 5' AAACGTGCCGATACAGCTCGAGAAC 3'

**PDE10A sgRNA-3**

____20nt-sgRNA3_____

5' CACCGGAACGATTTCCAAGAGGTAC 3'

3' CCTTGCTAAAGGTTCTCCATGCAAA 5'

PDE10A-gRNA3-top: 5' CACCGGAACGATTTCCAAGAGGTAC 3'

PDE10A-gRNA3-bottom: 5' AAACGTACCTCTTGGAAATCGTTCC 3'

The oligonucleotides above were synthesized with 5’-end phosphorylated ends (Life Technologies) to facilitate cloning into plasmid pSpCas9(BB)2A-GFP (Addgene #48138) as described (1). It is worth noticing that this backbone CRISPR/Cas9 plasmid does not confer resistance to antibiotics; hence, Cas9 and GFP expression occur only transiently in transfected cells. This choice of plasmid was intentional, given that once Cas9-mediated genome editing occurs, it is permanent, and we deemed unnecessary and undesirable to select for cells stably expressing Cas9. The efficiency of indel for the two constructed plasmids, PDE10A-sgRNA2-spCas9(BB)2A-GFP and PDE10A-sgRNA3-spCas9(BB)2A-GFP, was confirmed by transfection in 293T cells and subsequent Surveyor assay analysis as described.^1^ Briefly, 48 h post-transfection of 293T cells with pSpCas9(BB)2A-GFP, PDE10A-sgRNA2-spCas9(BB)2A-GFP or PDE10A-sgRNA3-spCas9(BB)2A-GFP using Lipofectamine® 3000 Transfection Reagent (Life Technologies), genomic DNA was extracted with DNeasy Blood and Tissue kit (QIAGEN). Conditions for PCR with Herculase II Fusion DNA Polymerase (Agilent Technologies) and SURVEYOR® PLUS Mutation Detection Kit (Integrated DNA Technologies) were the same as described by Ran *et al*.^1^ Calculated indel efficiency of PDE10A sgRNA-3 based on Surveyor results was approximately 34%, while for sgRNA-2 was much lower but detectable by Sanger sequencing of gel-purified PCR product and Tracking of Indels by Decomposition analysis using TIDE (https://tide.nki.nl/#about).^2^

Because detection of PDE10 protein expression by western-blotting proved difficult with commercially available antibodies at the time this work was performed (including Genetex rabbit polyclonal anti-PDE10A cat# GTX118886), due to lack of specificity and many cross-reactivity bands, we devised a PCR-based approach to screen for successful genome editing by the CRISPR/Cas9 PDE10A sgRNAs. The predicted Cas9 cleavage sites for PDE10A sgRNA-2 and sgRNA-3 are 97-bp apart in exon 7; their simultaneous deletion would result in disruption of the PDE10A ORF and therefore functionally knockout PDE10 protein. Therefore, we co-transfected the two PDE10A sgRNA constructs (PDE10A-sgRNA2-spCas9(BB)2A-GFP and PDE10A-sgRNA3-spCas9(BB)2A-GFP) in OV-90 cells, and then performed genomic DNA (gDNA)-based PCR to screen for the 97-bp deletion in the isolated cell clones. The workflow for PDE10A knockout is described in Figure S2. A primer pair (gRNA2-7i-fw3 and gRNA2-7i-rv3; Table S4) was designed to flank the regions immediately upstream of PDE10A sgRNA-3 and downstream of sgRNA-2, resulting in PCR amplification of a 549bp DNA fragment in wild-type / parental cells, while simultaneous successful cleavage by *S. pyogenes* Cas9 for both PDE10A sgRNAs was predicted to result in a PCR product of ~450bp. This reduction in size could be easily detected in 2% agarose gels and was used for the screening of PDE10A knockout clones (Fig. S2B). Hence, OV-90 cells were co-transfected using Lipofectamine® 3000 and 48 h post-transfection, cells were sorted to enrich the GFP+ population and single-cell cloned. Three weeks after GFP+ sorting, single-cell derived clones were expanded for further analyses. Clones with promising PCR profile were further analyzed by Sanger DNA sequencing to confirm the homozygous deletion of the PDE10A gene at exon and disruption of its ORF as predicted. OV-90 PDE10A knockout (KO) clones and a clone that preserved intact PDE10A ORF (called WT, or wild-type) were selected for phenotypic analyses. The strategy to knockout PDE10A in SKOV3 cells was similar, except that we used western-blotting with a knockout validated PDE10A monoclonal antibody from Abcam (ab227829) which was released in early 2019.

**TCGA data analysis:** PDE10A mRNA expression and patient clinical survival data for ovarian cystadenocarcinoma patients of The Cancer Genome Atlas (TCGA) was downloaded using the cBioPortal.^3,4^ Overall and disease-free survival analysis was performed using GraphPad Prism 8 to obtain Kaplan-Meyer survival curves and log-rank comparisons.

Co-expression data for PDE10A was retrieved from the cBioportal (Table S5), which provided a list of Spearman correlation values between PDE10A and all genes in the RNAseq V2 RSEM. Pathway analysis was performed for the top 2,000 genes positively correlated with PDE10A (Spearman r>0.24 and q-value<0.0001), and the top 1,125 genes negatively correlated with PDE10A (Spearman r<-0.2 and q-value< 0.0025). Molecular pathways were investigated with MSigDB as described below (Tables S6-11). Heatmap for pathways/genes of interest were generated using ClustVis (https://biit.cs.ut.ee/clustvis/).^5^

Additionally, the mRNA comparison tool in the cBioportal was used to compare TCGA ovarian tumors (Firehose Legacy, 307 samples with mRNA data; RNA Seq V2) with PDE10A^HIGH^ (Z>1.5; n=15) *vs.* PDE10A^LOW^ (Z<-1.5; n=13) expression and obtain log2-fold changes and adjusted p-values for all measured genes in the RNAseq dataset. These data were subsequently input into iPathway Guide (Advaita Bio) for impact data analysis as described below, using thresholds of log2-fold change ±0.6 and adjusted p-value < 0.1. In this experiment, 1,510 differentially expressed (DE) genes were identified out of a total of 19,331 genes with measured expression (Table S12).

**RNA sequencing:** mRNA-sequencing was performed on the Illumina NextSeq500 as described by the manufacturer (Illumina Inc., San Diego, CA) by the Heflin Center for Genomic Science Core Laboratories at the University of Alabama in Birmingham. Briefly, RNA quality was assessed using the Agilent 2100 Bioanalyzer. RNA with a RNA Integrity Number (RIN) of ≥7.0 was used for sequencing library preparation. RNA passing quality control was converted to a sequencing ready library using the NEBNext Ultra II Directional RNA library kit as per the manufacturer’s instructions (NEB, Ipswich, MA). The cDNA libraries were quantitated using qPCR in a Roche LightCycler 480 with the Kapa Biosystems kit for Illumina library quantitation (Kapa Biosystems, Woburn, MA) prior to cluster generation. Cluster generation was performed according to the manufacturers recommendations for onboard clustering (Illumina, San Diego, CA). We generated between 30-35 million paired end 75bp sequencing reads per sample for transcript level abundance. STAR (version 2.7.3a) was used to align the raw RNA-Seq fastq reads to the reference genome (GRCh38 p13, Release 32) from Gencode using parameters --outReadsUnmapped Fastx --outSAMtype BAM SortedByCoordinate --outSAMattributes All.^6^ Following alignment, HTSeq-count version 0.11.3 was used to estimate transcript abundances using parameters -m union -r pos -t exon -i gene_id -a 10 -s no -f bam.^7^ Normalization and differential expression were then applied to the count files using DESeq2 version 1.26.0 following their vignette.^8^ STAR alignment statistics, normalized counts, differential gene expression and pathway analysis data are provided in Supplemental Tables S14-S22.

**Pathway Analysis:** First, DESeq2 differentially expressed genes (log2-fold change ±2; adjusted p-value < 0.05) molecular pathways (KEGG, Hallmark and Reactome genesets) were investigated with MSigDB in the UC San Diego and Broad Institute GSEA portal (https://www.gsea-msigdb.org/gsea/index.jsp).^9–11^ Differential gene expression data (log2-fold change and adjusted p-value) obtained using DESeq2 were input into iPathway Guide (Advaita Bio) of impact analysis analyzed in the context of pathways obtained from the Kyoto Encyclopedia of Genes and Genomes (KEGG) database (Release 96.0+/11-21, Nov 20).^12–16^ Impact analysis uses two types of evidence: i) the over-representation of differentially expressed (DE) genes in a given pathway and ii) the perturbation of that pathway computed by propagating the measured expression changes across the pathway topology. These aspects are captured by two independent probability values, pORA and pAcc, that are then combined in a unique pathway-specific p-value. The underlying pathway topologies, comprised of genes and their directional interactions, are obtained from the KEGG database.^15,17–19^ The first probability, pORA, expresses the probability of observing the number of DE genes in a given pathway that is greater than or equal to the number observed, by random chance.^20,21^ The second probability, pAcc, is calculated based on the amount of total accumulation measured in each pathway.

**Real-time quantitative polymerase reaction (RT-qPCR) for PDE10A:** Fresh frozen normal ovary and human ovary tumor tissues were pulverized in liquid nitrogen with mortar and pestle. Total RNA was isolated with AllPrep® DNA/RNA/miRNA Universal Kit (Qiagen), using QIAshredder (Qiagen) for complete tissue homogeneization prior to RNA isolation and in-column DNase treatment, as described per manufacturer’s instructions. Total RNA from ovarian cancer cell lines was isolated using TRIzol® Reagent (ThermoFisher Scientific) followed by TURBO DNA-free^TM^ kit (ThermoFisher Scientific) as per manufacturer’s instructions. One microgram of DNase-treated RNA was used for cDNA synthesis with High-Capacity cDNA Reverse Transcription Kit (ThermoFisher Scientific), using random primers as per manufacturer’s instructions. RT-qPCR primers to amplify human PDE10A mRNA expression and various housekeeping genes are shown in Table S3. RT-qPCR reactions were performed using SsoAdvanced™ Universal SYBR® Green Supermix using the CFX96 Touch Real-Time PCR Detection System (BioRad) with the following cycling conditions: denaturation for 10 min at 95 ^o^C; 50 cycles of denaturation at 95 ^o^C for 10 min and annealing / extension at 60 ^o^C for 1 min.

**Cell proliferation:** Cells were seeded in 24-well tissue culture plates and incubated for 4 days (5,000 cells / well; 4 replicate wells per cell line per day). Nuclei were fluorescently labeled with NucBlue (ThermoFisher Scientific) for 10 minutes at 37^o^C. Cell counts were generated by measuring number of fluorescent nuclei using the Celigo Imaging Cytometer (Nexcelom). Growth ratio was calculated by comparing daily cell counts to baseline (day 0).

**Colony formation assay**: Cells were seeded in 6-well tissue culture plates (200 or 400 cells / well) in complete cell growth medium and, when applicable, treated with DMSO or compounds at indicated concentrations for 12 days (37°C, 5% CO_2_). Colonies were stained with Crystal Violet, then imaged using Chemidoc^TM^ Gel Imaging System (BioRad), and counted using the Nikon elements software system.

**Cell cycle analysis:** Cells rigorously kept in logarithmic growth for one week were seeded in 6-well plates (100,000 cells / well) in triplicate and incubated at 37^o^C for 24 or 48 h in the presence of vehicle or drug. At the end of incubation, cells were detached with trypsin, washed with ice-cold 1x Dulbecco Phosphate Buffer Saline (1x DPBS) (ThermoFisher Scientific), fixed with 70% ethanol overnight at -20^o^C. Post-fixation, cells were washed with 1xDPS and stained with undiluted Propidium Iodide (PI)/RNase Staining Solution (Cell Signaling) for 30 minutes at 37^o^C and analyzed by flow-cytometry on a BD-FACS Canto^TM^ (BD Biosciences). The percentage of cell population in each phase of the cell cycle was calculated using Mod Fit LT software (Verity Software House, Topsham, ME).

**Migration and invasion assays:** Cells were plated (50,000 / well; 4 replicate wells per cell line) in the top chamber of Cultrex 96-well migration and BME invasion plates (Trevigen) in culture medium lacking serum. The bottom chambers contained medium with 10% FBS as a chemoattractant. After 24 h of incubation, the medium was removed from top and bottom chambers, and wells were washed as described by the manufacturer. To adequately detach migrated / invaded cells, TrypleLE^TM^ Express (ThermoFisher Scientific) containing NucBlue was added to the bottom chambers and allowed to incubate with the top chamber for 15 min. Complete medium was then added to neutralize trypsin, and detached cells were sedimented to the bottom of the wells by centrifugation (1,000 x g for 5 minutes). Cells were then imaged and counted using the Celigo Imaging Cytometer (Nexcelom) by measuring the number of fluorescent nuclei. Percent of migration or invasion relative to parental cells was calculated after normalizing to seeding values.

**Cell treatments and lysis for Western-blot:** Cells were plated (2.5 x 10^5^ cells / well) in 6-well tissue culture plates. Drug treatments are indicated in the figure legends when applicable. At the end of the assay, attached cells were rinsed with ice-cold 1x DPBS, followed by cell lysis with NP-40 Lysis Buffer (Boston Bioproducts Inc.) containing 1x Halt^TM^ Protease and Phosphatase Inhibitor Single-Use Cocktail (ThermoFisher Scientific). For assays that evaluated PARP cleavage after drug incubation, floating cells in the supernatant medium were collected by centrifugation and added to the lysate pool. Cell lysates were incubated on ice for 5-10 minutes and centrifuged at 15,000 x g at 4°C for 10 minutes. Protein determination was performed with Pierce™ BCA Protein Assay Kit (ThermoFisher Scientific).

**Western-blot:** Cell lysates (30 μg of protein / lane) were separated by SDS-PAGE in polyacrylamide gel (10-12%) followed by electrophoretic transfer to nitrocellulose paper. Membranes were then blocked with 5% milk in Tris-Buffered Saline (TBS) containing 0.1% Tween 20 (TBS-T). Membranes were incubated overnight with primary antibody at 4°C on an orbital shaker. Membranes were then washed three times in TBS-T before adding secondary antibody and incubating for 1 hour on an orbital shaker. Three subsequent TBS-T washes were performed then membranes were developed using SuperSignal West or Femto Chemiluminescent Substrates (Thermo Scientific) and a Chemi Doc Imaging System (BioRad). Quantitation of western blots was performed using ImageLab 6.0.1 software (BioRad).

**Phosphodiesterase activity assay:** Cancer cells or mouse whole brain were homogenized in buffer containing 20 mM HEPES (pH 7.4), 1 mM EDTA, 0.2 mM EGTA, 150 mM NaCl, 20% sucrose, Halt^TM^ Protease & Phosphatase Inhibitor Cocktail (Thermo Scientific, Rockford, IL) and 1% Triton X-100. After a 30-minute rotation at 4 °C, cell debris were pelleted (10-minute centrifugation at 20,000 g, 4^o^C), and soluble extracts were then subjected to IP using 40 μl Protein A/G Sepharose (Santa Cruz Biotech, Santa Cruz, CA) and 5 μg of either anti-PDE10A mouse monoclonal antibody (SC-515023; Santa Cruz Biotech, Santa Cruz, CA) or normal mouse IgG as control. After incubation for 16 h (overnight) at 4 °C, the resin was washed 3 times with 500 μl lysis buffer, and protein recovered in the IP pellets was then detected by PDE activity assays. Cyclic AMP-PDE activity was measured following a protocol described previously with minor modifications.^22^ In brief, samples were assayed in a reaction mixture of 200μl containing 40 mM Tris-HCl (pH 7.4), 10 mM MgCl_2_, 1.34 mM β-mercaptoethanol, 1 μM cAMP, and 0.1 μCi [^3^H]cAMP (Perkin Elmer, Waltham, MA) for 60 min at 37 °C followed by heat inactivation in a boiling water bath for 1 min. The PDE reaction product 5′-AMP was then hydrolyzed by incubation of the assay mixture with 50 μg *Crotalus atrox* snake venom (Sigma-Aldrich, St. Louis, MO) for 20 min at 37 °C; the resulting adenosine was afterwards separated by anion exchange chromatography on 1 ml of AG1-X8 resin (Bio-Rad Laboratories, Hercules, CA) and quantitated by scintillation counting. PDE10A activity was defined as the fraction of total cAMP-PDE activity inhibited by 100 nM of the PDE10A-selective inhibitor Pf-2545920 compared to solvent / DMSO control.

**References:**

1. Ran FA, Hsu PD, Wright J, Agarwala V, Scott DA, Zhang F. Genome engineering using the CRISPR-Cas9 system. *Nat Protoc*. 2013;8(11):2281-2308. doi:10.1038/nprot.2013.143

2. Brinkman EK, Chen T, Amendola M, Van Steensel B. Easy quantitative assessment of genome editing by sequence trace decomposition. *Nucleic Acids Res*. 2014. doi:10.1093/nar/gku936

3. Gao J, Aksoy BA, Dogrusoz U, et al. Integrative analysis of complex cancer genomics and clinical profiles using the cBioPortal. *Sci Signal*. 2013;6(269):1-20. doi:10.1126/scisignal.2004088

4. Cerami E, Gao J, Dogrusoz U, et al. The cBio Cancer Genomics Portal: An open platform for exploring multidimensional cancer genomics data. *Cancer Discov*. 2012;2(5):401-404. doi:10.1158/2159-8290.CD-12-0095

5. Metsalu T, Vilo J. ClustVis: A web tool for visualizing clustering of multivariate data using Principal Component Analysis and heatmap. *Nucleic Acids Res*. 2015;43(W1):W566-W570. doi:10.1093/nar/gkv468

6. Dobin A, Davis CA, Schlesinger F, et al. STAR: Ultrafast universal RNA-seq aligner. *Bioinformatics*. 2013. doi:10.1093/bioinformatics/bts635

7. Anders S, Pyl PT, Huber W. HTSeq-A Python framework to work with high-throughput sequencing data. *Bioinformatics*. 2015. doi:10.1093/bioinformatics/btu638

8. Love MI, Huber W, Anders S. Moderated estimation of fold change and dispersion for RNA-seq data with DESeq2. *Genome Biol*. 2014;15(12). doi:10.1186/s13059-014-0550-8

9. Liberzon A, Subramanian A, Pinchback R, Thorvaldsdóttir H, Tamayo P, Mesirov JP. Molecular signatures database (MSigDB) 3.0. *Bioinformatics*. 2011;27(12):1739-1740. doi:10.1093/bioinformatics/btr260

10. Mootha VK, Lindgren CM, Eriksson KF, et al. PGC-1α-responsive genes involved in oxidative phosphorylation are coordinately downregulated in human diabetes. *Nat Genet*. 2003. doi:10.1038/ng1180

11. Subramanian A, Tamayo P, Mootha VK, et al. Gene set enrichment analysis: A knowledge-based approach for interpreting genome-wide expression profiles. *Proc Natl Acad Sci U S A*. 2005. doi:10.1073/pnas.0506580102

12. Draghici S, Khatri P, Tarca AL, et al. A systems biology approach for pathway level analysis. *Genome Res*. 2007. doi:10.1101/gr.6202607

13. Donato M, Xu Z, Tomoiaga A, et al. Analysis and correction of crosstalk effects in pathway analysis. *Genome Res*. 2013. doi:10.1101/gr.153551.112

14. Ahsan S, Drăghici S. Identifying significantly impacted pathways and putative mechanisms with iPathwayGuide. *Curr Protoc Bioinforma*. 2017. doi:10.1002/cpbi.24

15. Kanehisa M, Goto S. KEGG: Kyoto Encyclopedia of Genes and Genomes. *Nucleic Acids Res*. 2000. doi:10.1093/nar/28.1.27

16. Kanehisa M, Goto S, Kawashima S, Nakaya A. Thed KEGG databases at GenomeNet. *Nucleic Acids Res*. 2002. doi:10.1093/nar/30.1.42

17. Kanehisa M, Goto S, Furumichi M, Tanabe M, Hirakawa M. KEGG for representation and analysis of molecular networks involving diseases and drugs. *Nucleic Acids Res*. 2009. doi:10.1093/nar/gkp896

18. Kanehisa M, Goto S, Sato Y, Furumichi M, Tanabe M. KEGG for integration and interpretation of large-scale molecular data sets. *Nucleic Acids Res*. 2012. doi:10.1093/nar/gkr988

19. Kanehisa M, Goto S, Sato Y, Kawashima M, Furumichi M, Tanabe M. Data, information, knowledge and principle: Back to metabolism in KEGG. *Nucleic Acids Res*. 2014. doi:10.1093/nar/gkt1076

20. Drǎghici S, Khatri P, Martins RP, Ostermeier GC, Krawetz SA. Global functional profiling of gene expression. *Genomics*. 2003. doi:10.1016/S0888-7543(02)00021-6

21. Draghici S. *Statistics and Data Analysis for Microarrays Using R and Bioconductor*.; 2016. doi:10.1201/b11566

22. Xie M, Blackman B, Scheitrum C, et al. The upstream conserved regions (UCRs) mediate homo-and hetero-oligomerization of type 4 cyclic nucleotide phosphodiesterases (PDE4s). *Biochem J*. 2014;459(3):539-550. doi:10.1042/BJ20131681
